# Bi-directional cell-pericellular matrix interactions direct stem cell fate

**DOI:** 10.1038/s41467-018-06183-4

**Published:** 2018-10-03

**Authors:** Silvia A. Ferreira, Meghna S. Motwani, Peter A. Faull, Alexis J. Seymour, Tracy T. L. Yu, Marjan Enayati, Dheraj K. Taheem, Christoph Salzlechner, Tabasom Haghighi, Ewa M. Kania, Oommen P. Oommen, Tarek Ahmed, Sandra Loaiza, Katarzyna Parzych, Francesco Dazzi, Oommen P. Varghese, Frederic Festy, Agamemnon E. Grigoriadis, Holger W. Auner, Ambrosius P. Snijders, Laurent Bozec, Eileen Gentleman

**Affiliations:** 1 Centre for Craniofacial and Regenerative Biology, King’s College London, London, SE1 9RT UK; 2 Protein Analysis and Proteomics Platform, The Francis Crick Institute, London, NW1 1AT UK; 3 Ludwig Boltzmann Cluster for Cardiovascular Research at the Center for Biomedical Research, Medical University of Vienna, Vienna, Austria; 4 Bioengineering and Nanomedicine Lab, Faculty of Biomedical Sciences and Engineering, Tampere University of Technology and BioMediTech Institute, 33720 Tampere, Finland; 5 Biomaterials and Tissue Engineering, Eastman Dental Institute, University College London, London, WC1X 8LD UK; 6 Cancer Cell Protein Metabolism Group, Department of Medicine, Imperial College London, London, W12 0NN UK; 7 Department of Haemato-Oncology, Rayne Institute, King’s College London, London, SE5 9NU UK; 8 Department of Chemistry, Ångström Laboratory, Science for Life Laboratory, Uppsala University, SE-75121 Uppsala, Sweden; 9 Tissue Engineering and Biophotonics, King’s College London, London, SE1 9RT UK; 10 Faculty of Dentistry, University of Toronto, 124 Edward Street, Toronto, Toronto, ON M5G 1G6 Canada

**Keywords:** Stem-cell differentiation, Biomaterials - cells

## Abstract

Modifiable hydrogels have revealed tremendous insight into how physical characteristics of cells’ 3D environment drive stem cell lineage specification. However, in native tissues, cells do not passively receive signals from their niche. Instead they actively probe and modify their pericellular space to suit their needs, yet the dynamics of cells’ reciprocal interactions with their pericellular environment when encapsulated within hydrogels remains relatively unexplored. Here, we show that human bone marrow stromal cells (hMSC) encapsulated within hyaluronic acid-based hydrogels modify their surroundings by synthesizing, secreting and arranging proteins pericellularly or by degrading the hydrogel. hMSC’s interactions with this local environment have a role in regulating hMSC fate, with a secreted proteinaceous pericellular matrix associated with adipogenesis, and degradation with osteogenesis. Our observations suggest that hMSC participate in a bi-directional interplay between the properties of their 3D milieu and their own secreted pericellular matrix, and that this combination of interactions drives fate.

## Abstract (web summary)

3D hydrogels have provided information on the physical requirements of stem cell fate, but the contribution of interactions with the pericellular environment are under-explored. Here the authors show that pericellular matrix secreted by human bone marrow stromal cells (hMSC) embedded in a HA-based hydrogel contribute to hMSC fate.

## Introduction

Regenerative therapies that combine stem cells with materials offer tremendous clinical promise, yet, controlling differentiation and tissue formation remain a pressing challenge. In addition to soluble factors, physical characteristics of the extracellular milieu are known to direct lineage specification^1^, however, how cell-extracellular matrix (ECM) interactions drive this process in in vivo-like environments remains incompletely understood. In 3D hydrogels^2,3^, a stem cell’s ability to probe hydrogel stiffness^4^ and degrade its surroundings^5^ regulates fate. However, such findings are complicated in hydrogels with time-dependent properties that better mimic the native ECM^6^. Hydrogels that stiffen or soften in a controlled manner^7,8^, that undergo stress stiffening^9^, or are viscoelastic^10–13^ have revealed a role for dynamic changes in hydrogel physical properties in guiding stem cell fate.

However, cells do not passively respond to signals delivered to them, whether they are static or dynamic. Instead, many cell types actively modify their local environment by secreting a proteinaceous ECM and degrading their surroundings to suit their needs. This is apparent in the epidermis, where cell–ECM interactions reciprocally regulate the stem cell niche^14,15^. Disrupting this balance, as occurs in epidermolysis bullosa, a family of skin blistering disorders in which cells fail to deposit type VII collagen, demonstrates the importance of bi-directional interactions in tissue maintenance. Reciprocal cell-ECM interactions have also been described in the field of biomaterials, where cells quickly “compatibilize” non-adhesive surfaces by secreting/assembling a proteinaceous matrix which they actively probe^16^, even in the absence of serum proteins^17^. The role of bi-directional interactions in 3D hydrogels are less well studied, and while secreted ECM has been hypothesized to influence cell response^4,18,19^, how it directs encapsulated cells remains relatively unexplored.

Here, we show that when encapsulated within hyaluronic acid (HA)-based hydrogels, hMSC quickly modify their surroundings via protein secretion and/or matrix degradation. These cell-mediated local modifications impact hMSC fate, with secretion of a proteinaceous pericellular matrix driving adipogenesis and degradation of the hydrogel matrix promoting osteogenesis. Our findings suggest that hydrogel physical properties may not direct fate in isolation, but rather impact how hMSC modulate their pericellular surroundings, which in turn directs differentiation.

## Results

### Encapsulated hMSC form a proteinaceous pericellular matrix

**Fig. 1:**
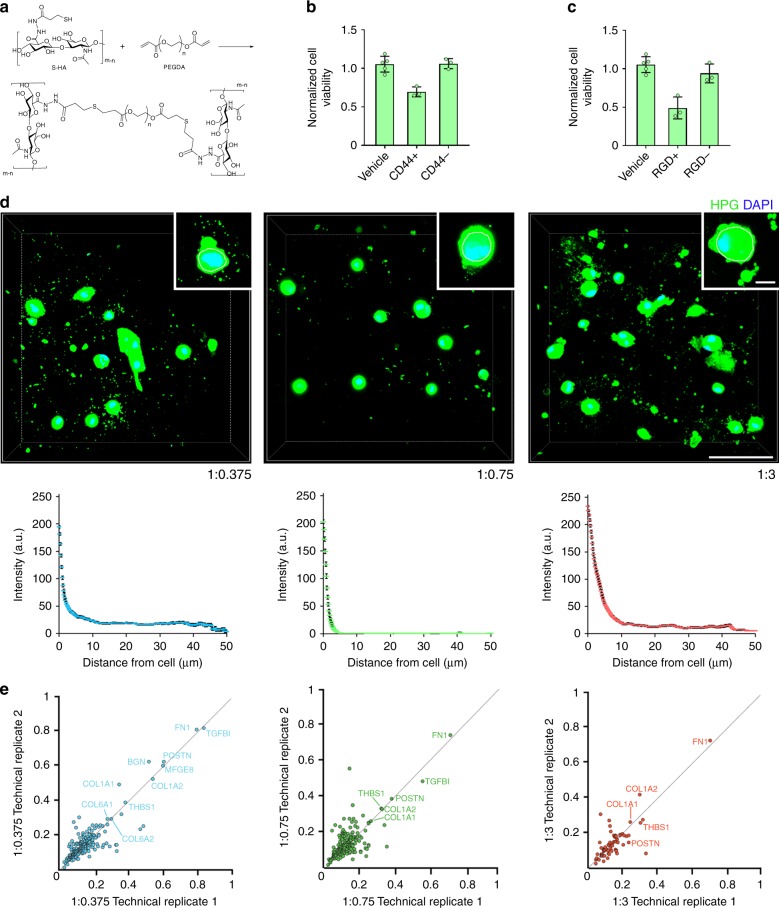
hMSC within S-HA-PEGDA hydrogels synthesize and secrete proteins pericellularly. **a** Reaction scheme for hydrogel formation. Thiol-modified hyaluronic acid (S-HA) cross-links with poly(ethylene glycol) diacrylate (PEGDA) to form a hydrogel via a Michael addition. **b** Viability of hMSC encapsulated in 1:0.75 hydrogels treated with an anti-CD44 (CD44+) antibody or an isotype control (CD44−) for 24 h and normalized to vehicle controls (*n* ≥ 3). **c** Viability of hMSC encapsulated in 1:0.75 hydrogels treated for 24 h with RGD sequence-containing peptides (RGD+) or scrambled peptides (RGD−) and normalized to vehicle controls (*n* ≥ 3). In **b** and **c** plots show means ± s.d. **d** Representative micrographs showing fluorescence labeling of the methionine analogue l-homopropargylglycine (HPG, green) in hMSC-laden 1:0.375, 1:0.75, and 1:3 hydrogels after 72 h in culture. The cell membrane, as determined by differential interference contrast (DIC) imaging, is outlined in white. Scale bar = 100 µm, inset = 10 µm. Plots show mean intensity of the fluorescence signal (±s.e.m.) as a function of distance from the cell membrane in 1:0.375 (blue dashes), 1:0.75 (green dashes), and 1:3 (red dashes) hydrogels and were generated from 40 profile plots collected from 30 cells per condition. **e** Fraction of SILAC heavy-labeled proteins in decellularized 1:0.375 (blue circles), 1:0.75 (green circles), and 1:3 (red circles) hydrogels cultured for 72 h. Scatterplots show two technical replicates (*H/L* count ≥ 3) for each hydrogel composition. Gene names for ECM proteins showing high levels (>40%) of SILAC incorporation are highlighted in each panel

To study the role of cell-secreted ECM in regulating hMSC fate in 3D, we utilized hydrogels based on a well-described Michael addition between thiol-modified HA (S-HA) and poly(ethylene glycol) diacrylate (PEGDA) (Fig. 1a)^20^. S-HA-PEGDA hydrogels form quickly under mild conditions, allowing cell encapsulation. They also offer insight into the role of cell-secreted ECM in directing fate, because like un-modified PEG, they provide no sites for integrin-mediated interactions. However, unlike in PEG where the lack of adhesive motifs can prompt anoikis^21^, HA interactions with surface receptors, such as CD44 and RHAMM^22^, allow for long-term cell viability^23^, limiting integrin-mediated interactions to those with the cells’ own secreted ECM. Moreover, like other modifiable hydrogels, S-HA-PEGDA’s physical properties can be tuned over a wide range^8^.

By holding the concentration of S-HA constant and varying the concentration of PEGDA (described as weight ratios, 1:relative weight PEGDA), we formed hydrogels that ranged from being primarily composed of S-HA to PEGDA-dominated hydrogels (Supplementary Table 1). We then carried out standard characterization techniques and found that S-HA-PEGDA hydrogels undergo expected^24^ PEGDA concentration-dependent swelling (Supplementary Fig. 1). Similarly, treatment with hyaluronidase results in PEGDA concentration-dependent degradation (Supplementary Fig. 2), confirming that HA remains integral to the hydrogel network and that the thiol-modification does not preclude enzymatic degradation. Atomic force microscopy (AFM)-based indentation measurements 72 h after cross-linking showed that Young’s modulus (*E*) increased with increasing PEGDA concentration until the molar ratio of acrylate exceeded that of thiols required for ideal cross-linking, after which adding more PEGDA decreased stiffness^25^. Over time and in line with enzyme-mediated degradation (in the presence of serum) and inherent degradation via ester hydrolysis^8^, hydrogels softened and differences in *E* among compositions were attenuated (Supplementary Fig. 3). Although not explicitly designed into the system, these time-dependent behaviors were in line with those observed in biological systems which self-modify over days to weeks^26^.

We then encapsulated hMSC in S-HA-PEGDA hydrogels and observed that they remained viable, but exhibited limited proliferation over 4 weeks (Supplementary Fig. 4), as previously described^9,27^. Encapsulated hMSC also adopted round morphologies (Supplementary Fig. 5) regardless of PEGDA concentration, in keeping with the lack of adhesive motifs within S-HA-PEGDA hydrogels. Quantification by flow cytometry of free thiols on hMSC’s surfaces^28^ after labeling with a maleimide-modified Alexa Fluor showed no differences compared to N-ethylmaleimide-treated controls (Supplementary Fig. 6), confirming that few if any covalent bonds were possible between hMSC and hydrogels. We then blocked cells’ interactions with HA using an anti-CD44 antibody and observed a quick (24 h) drop in viability compared to treatment with isotype controls (Fig. 1b). This confirmed HA’s role in promoting survival of encapsulated cells in the absence of integrin-mediated interactions. Nevertheless, when we added peptides containing an RGD sequence, which block many integrin-mediated interactions, we observed a surprising similar reduction in viability (Fig. 1c). Therefore, while hMSC-HA interactions via CD44 had an expected role, integrin-mediated interactions also appeared to have a quick, unexpected role in maintaining viability, even though hydrogels had not been modified with adhesive motifs.

To understand how integrin-mediated interactions could have influenced viability, we next labeled proteins synthesized by hMSC over the first 72 h after encapsulation using a non-canonical amino acid tagging technique, which substitutes the canonical amino acid methionine with a non-canonical analogue that contains a bio-orthogonal functional group^29^. Using a simple click chemistry to fluorescently identify the incorporated label, this allowed us to image intracellular proteins as well as secreted proteins retained in the hydrogel surrounding hMSC. Images of labeled proteins showed that hMSC in 1:0.375 and 1:3 hydrogels assembled an extensive proteinaceous pericellular matrix around themselves, while in 1:0.75 hydrogels, the pericellular matrix appeared to be more limited (Fig. 1d). Quantification of the mean intensity of the signal of labeled proteins in radii measured from the cell membrane showed that in 1:0.375 and 1:3 hydrogels, secreted proteins were detectable more than 40 µm from the cell surface, but in 1:0.75 hydrogels, we detected little to no signal beyond ~5 µm. These observations show that while hMSC secrete proteins under all conditions, hydrogel composition influences secreted proteins’ density and distribution in the pericellular space.

To better understand the composition of this secreted matrix, we next performed a stable isotope labeling with amino acids in cell culture (SILAC) experiment to identify proteins produced by hMSC post-encapsulation. SILAC media contains heavy isotope labeled arginine and lysine, which are metabolically incorporated into newly synthesized proteins. We then decellularized hydrogels and applied an “in-hydrogel digestion” method that allowed us to use mass spectrometry to determine the fraction of more than 1100 proteins remaining in hydrogels that contained the heavy label (Fig. 1e, Supplementary Fig. 7 and Supplementary Data 1). ECM proteins including fibronectin, collagens and periostin, among others, showed high levels (>40%) of incorporation within all hydrogel compositions.

Taken together, these observations provide unequivocal evidence that hMSC synthesize, secrete and assemble a proteinaceous pericellular matrix around themselves post-encapsulation. They also show that secreted proteins have a role in maintaining cell viability and suggest that when adhesive motifs are not available in 3D culture, hMSC synthesize them.

### Encapsulated hMSC modulate their pericellular stiffness

**Fig. 2:**
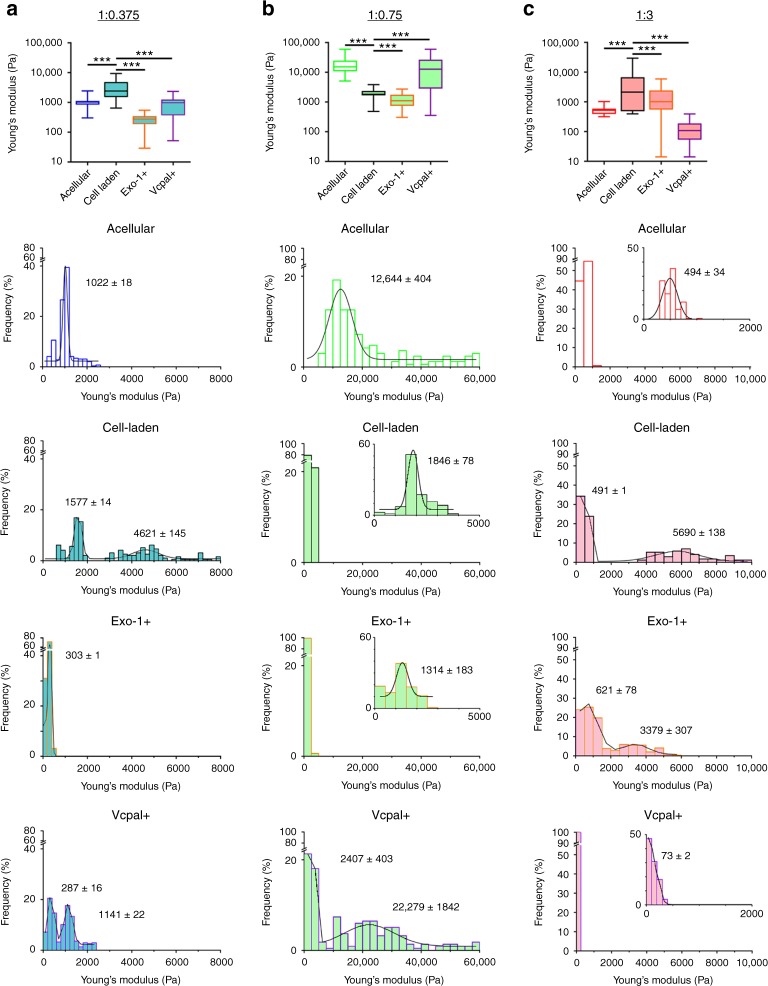
Hydrogel stiffness is modified by encapsulated hMSC. Young’s modulus (*E*, Pa) of acellular and cell-laden **a** 1:0.375, **b** 1:0.75, and **c** 1:3 S-HA-PEGDA hydrogels evaluated by atomic force microscopy. Box plots (top row) showing 1st/3rd quartiles (bounds of box), high/low values (whiskers), and median (central line) *E*. A Mann–Whitney test (two-tailed) was used to assess statistical differences (***p < 0.001). Histograms show distributions of *E* on acellular and hMSC-laden hydrogels cultured under standard conditions or treated with 75 μM Exo-1 or 100 μM Vcpal for 72 h. Histograms are overlaid with fitted normal distributions, with centre ± standard error. Insets show distributions and fitted normal distributions in designated areas of the datasets. In all compositions, the presence of hMSC significantly affected the distribution of *E* (see Supplementary Table 2 for *n* values and statistical analyses of distributions of *E*)

As hMSC synthesized and secreted proteins after encapsulation, we next asked what effect this had on hydrogel physical properties. AFM is particularly insightful in this context because by adapting the cantilever with a bead, we can probe *E* at the scale of a cell within live cultures. In 1:0.375 hydrogels, the presence of encapsulated hMSC mediated a significant increase in *E* after 3 days in culture (Fig. 2a). Cell-mediated effects were also evident in 1:3 hydrogels (Fig. 2c), where *E* increased by nearly an order of magnitude. Increases in *E* were not associated with either inherent or cell-mediated changes in bulk hydrogel dimensions (Supplementary Fig. 8), but rather accompanied by bi-modal distributions of *E* (Fig. 2, histograms). Unsupervised peak fitting identified one value similar to that of acellular controls and another that was stiffer. Treating cultures with Exo-1, an inhibitor of vesicular trafficking between the ER and Golgi^30^, and thus a protein secretion inhibitor, had no detectable effect on cellular metabolism (Supplementary Fig. 9). However, in 1:0.375 and 1:3 hydrogels, Exo-1 abolished (1:0.375) or significantly altered (1:3, Supplementary Table 2) the bi-modal distributions and effected a significant decrease in *E* (Fig. 2a, c). Drawing on observations from cartilage biomechanics, where differential stiffnesses have been detected between the pericellular and bulk extracellular space surrounding chondrocytes^31^, our observations suggest that in 1:0.375/1:3 hydrogels, hMSC secreted proteins to form a pericellular matrix that was stiffer than that of the bulk hydrogel.

In contrast to our observations in 1:0.375 and 1:3 hydrogels, in 1:0.75 hydrogels (Fig. 2b), the presence of hMSC mediated a significant decrease in *E*, and a bi-modal distribution was not evident. In 1:0.75 hydrogels, treatment with the hyaluronidase inhibitor Vcpal^32^ (which blocks hyaluronidase-mediated degradation of S-HA, Supplementary Fig. 9c) precipitated the appearance of a bi-modal distribution and a significant increase in *E*. These observations suggested that within 1:0.75 hydrogels, hMSC modulated their local environment predominantly via degradation.

Changes in *E* of cell-laden hydrogels have been reported, but often with chemical induction that stimulates extensive ECM formation and with higher cell densities^18,23,33,34^. Here, we used basal medium and relatively low cell densities, which allowed us to isolate how individual cells inherently modified their environment. Taken together, our observations suggest that within S-HA-PEGDA hydrogels, hMSC quickly modified their local environment, by degradation (to soften stiffer hydrogels) or secretion of proteins pericellularly (to locally stiffen softer hydrogels).

### Cell-mediated pericellular modifications drive hMSC fate

**Fig. 3:**
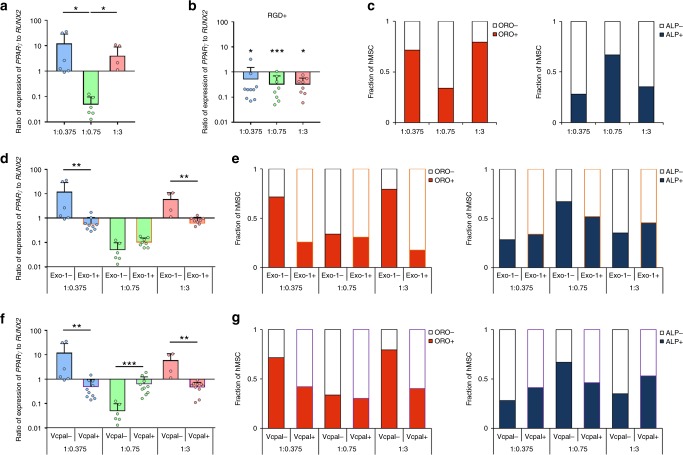
hMSC’s modifications to their pericellular environment directs fate. **a** Ratio of expression of *PPARγ* to *RUNX2* in hMSC encapsulated in hydrogels for 72 h and normalized to undifferentiated hMSC controls (mean + s.d.). Ratios were significantly higher in 1:0.375 and 1:3 (*p < 0.05) compared to 1:0.75 hydrogels (*n* ≥ 4). **b** Ratio of expression of *PPARγ* to *RUNX2* in hMSC after treatment with RGD sequence-containing peptides (RGD+) for 72 h (mean + s.d.). Ratios were not significantly different in hydrogels of different compositions (*n* ≥ 8), but for each composition, expression was significantly different than RGD− controls (1:0.375 and 1:3, *p < 0.05; 1:0.75, ***p < 0.001). **c** Fraction of hMSC that stained positively for Oil Red O (ORO+) or alkaline phosphatase (ALP+), as indicators of adipogenesis and osteogenesis, respectively, after 14 days in culture in a bi-potential osteogenic/adipogenic medium (*n* = 3300–1200 cells). **d** Ratio of expression of *PPARγ* to *RUNX2* in hMSC after treatment with 75 μM Exo-1 or **f** 100 μM Vcpal for 72 h (mean + s.d.). Fraction of hMSC that stained positively for ORO and ALP after treatment with either **e** 75 μM Exo-1 or **g** 100 μM Vcpal for 14 days (*n* = 3300–1200 cells). In **a** a Kruskal–Wallis followed by Dunn’s multiple comparison test was used to detect statistical significance. In **b**, **d**, and **f** a Mann–Whitney test (two-tailed) was used to compare treatment (RGD+, Exo-1+, Vcpal+) to control conditions within each hydrogel composition. In **c**, **e**, and **g** a Fisher’s exact test (two-sided) was used to compare treatment (Exo-1+, Vcpal+) to control conditions within each hydrogel composition (Supplementary Table 3). For representative images of ORO and ALP staining, see Supplementary Fig. 13

We next asked how cell-mediated changes in hydrogel properties affected hMSC differentiation, initially in the absence of chemical induction. Others have shown that HA promotes a bias towards the chondrogenic lineage^23,33^. And, indeed, pericellular staining for type II collagen and upregulation of chondrogenic genes *SOX9* and *COL2A1*, were evident in all compositions (Supplementary Fig. 10). However, examining markers for other fates yielded hydrogel composition-dependent differences in lineage specification. The ratio of expression of adipogenic and osteogenic transcription factors *PPARγ* and *RUNX2*, respectively, was significantly higher in hMSC encapsulated in 1:0.375 and 1:3 hydrogels compared to that in 1:0.75 hydrogels (Fig. 3a), suggesting that the former favored adipogenesis and the latter osteogenesis. This was supported by trends for upregulation of *BGLAP* (osteocalcin) in 1:0.75, and upregulation of *C/EBPα* (adipogenic transcription factor) in 1:0.375 and 1:3 hydrogels (Supplementary Fig. 11), which we confirmed by immunofluorescence staining (Supplementary Fig. 12). The addition of RGD sequence-containing peptides abolished differences in osteogenic/adipogenic gene expression (Fig. 3b and Supplementary Fig. 11), suggesting that integrin-mediated interactions with hMSC’s own secreted pericellular matrix biased their tendency towards different lineages. Longer-term culture in the presence of bi-potential osteogenic/adipogenic medium^35^ prompted hMSC to undergo terminal differentiation as determined by quantification of staining for alkaline phosphatase (ALP) and Oil Red O (ORO, Fig. 3c, Supplementary Fig. 13 and Supplementary Table 3). These observations confirmed that 1:0.375 and 1:3 hydrogels drove adipogenesis and 1:0.75 hydrogels prompted hMSC to undergo osteogenesis.

To better understand the role of cell-mediated modifications to the local pericellular environment in driving differentiation, we next inhibited protein secretion with Exo-1. Exo-1 abrogated adipogenesis in 1:0.375 and 1:3 hydrogels (Fig. 3d, e, Supplementary Fig. 13, 14, and Supplementary Table 3) and was observed concomitant with the loss of clear bi-modal distributions of *E* (Fig. 2a, c). These observations imply that formation of a proteinaceous pericellular matrix was necessary for adipogenesis in 1:0.375 and 1:3 S-HA-PEGDA hydrogels. Exo-1, however, did not affect the ratio of expression of *PPARγ* to *RUNX2* in 1:0.75 hydrogels and only subtly affected the fraction of ALP positive cells (Fig. 3d, e, Supplementary Fig. 13 and Supplementary Table 3), suggesting that protein secretion did not regulate osteogenesis in the 1:0.75 composition. Inhibiting hyaluronidase activity with Vcpal, on the other hand, abolished the upregulation of osteogenesis in 1:0.75 hydrogels (Fig. 3f, g, Supplementary Figs. 13, 14 and Supplementary Table 3) and coincided with the emergence of a bimodal distribution of *E* (Fig. 2b). This finding supports previous observations that degradation is a key mediator of hMSC lineage specification^5^. Vcpal, however, also significantly downregulated adipogenesis (Fig. 3f, g) in and softened 1:0.375 and 1:3 hydrogels (Fig. 2a, c), suggesting that hMSC may have to degrade their pericellular milieu to secrete ECM, as has been observed during native tissue remodelling^36^. And indeed, when we applied the non-canonical amino acid tagging technique to analyze the pericellular matrix that formed around hMSC after treatment with Vcpal, we observed less pericellular staining compared to controls (Supplementary Fig. 15).

To rule out a role of differential nutrient and/or growth factor diffusion in differentiation, we carried out fluorescence recovery after photobleaching, and failed to observe an effect of hydrogel composition on the diffusion of model molecules (Supplementary Fig. 16). Similarly, as HA fragments of different molecular weights have been reported to influence human embryonic stem cell self-renewal/differentiation^37^, we also treated hMSC with fragments from degraded hydrogels, but observed no effects on gene expression (Supplementary Fig. 17). Taken together, these findings suggest a bias in differentiation whereby cell-mediated formation of a proteinaceous pericellular matrix drives adipogenesis, and osteogenesis is driven by cell-mediated degradation of the matrix, which softens the hydrogel.

**Fig. 4:**
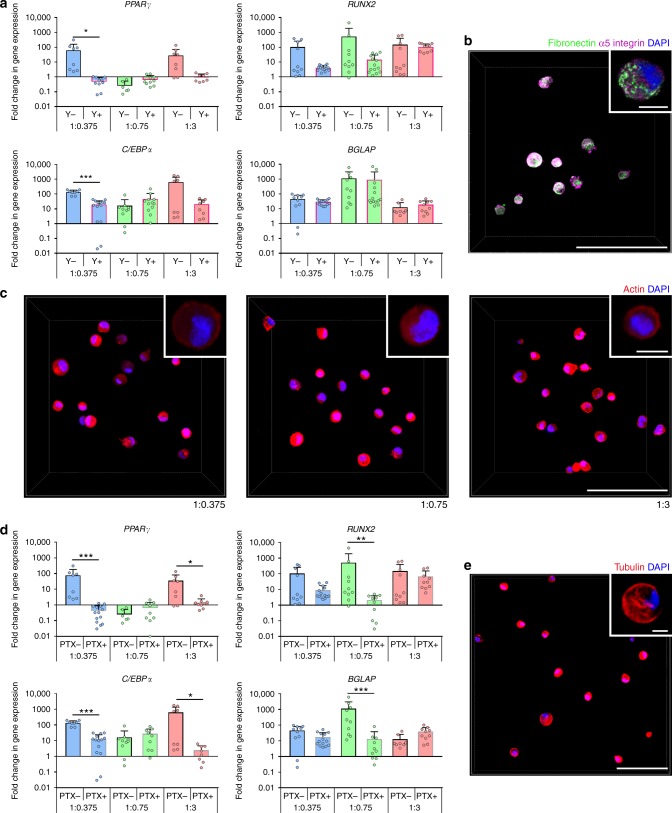
Y-27632 and paclitaxel impact osteogenic/adipogenic gene expression. **a** Gene expression analyses for markers of adipogenesis (*PPARγ* and *C/EPBα*) and osteogenesis (*RUNX2* and *BGLAP*) in hMSC cultured for 72 h with basal culture medium (−Y) or treated with 10 μM Y-27632 (+Y, *n* ≥ 8). Expression levels (mean + s.d.) are shown as fold change normalized to expression in undifferentiated hMSC (set to 1). **b** Representative micrograph of hMSC (5 × 10^6^ cells mL^−1^) encapsulated in a 1:0.75 hydrogel after 28 days showing fibronectin, α_5_ integrin, and DAPI. Fibronectin co-localized with punctate α_5_ integrin by Manders’ coefficients: Manders’ tM1 = 0.83 ± 0.18 and Manders’ tM2 = 0.84 ± 0.10 (*n* = 4, 25 cells). **c** Representative micrographs of hMSC within hydrogels after 72 h and stained with Phalloidin-TRITC (actin) and DAPI. **d** Gene expression analyses for markers of adipogenesis (*PPARγ* and *C/EPBα*) and osteogenesis (*RUNX2* and *BGLAP*) in hMSC cultured for 72 h with basal culture medium (−PTX) or treated with 50 nM paclitaxel (+PTX, *n* ≥ 8). Expression levels (mean + s.d.) are shown as fold change normalized to expression in undifferentiated hMSC (set to 1). **e** Representative micrographs of hMSC (5 × 10^6^ cells mL^−1^) within a 1:0.75 hydrogel for 72 h and stained for tubulin and DAPI. Scale bars in **b**, **c**, and **e** are 100 µm, and in insets = 10 µm. In **a** and **d** a Mann–Whitney test (two-tailed) was used to detect statistical significance, *p < 0.05, **p < 0.01, ***p < 0.001

Although hMSC could not form integrin-mediated interactions with the initial S-HA-PEGDA hydrogels, our data suggest that it was modified through a combination of degradative and secretive mechanisms. Although conflicting reports exist on the necessity of degradability in mechanotransduction-mediated hMSC differentiation in 3D^4,5,9^, there is more consensus on the need for adhesive ligands. In non-adhesive hydrogels, small molecule inhibitors of cytoskeletal tension have no effect on hMSC differentiation^19,38^, but when adhesive ligands are present, blocking induces a fate switch^5^ or abolishes differentiation^9^. When we treated cultures with the RhoA/ROCK inhibitor Y-27632, we observed a trend for an overall decrease in expression of genes indicative of both the osteogenic and adipogenic lineages, although this was not significant in many cases (Fig. 4a and Supplementary Fig. 18). This was in keeping with previous reports of hMSC response in hydrogels that undergo controlled stress-stiffening behaviour^9^. Our SILAC-based proteomic analysis revealed that hMSC encapsulated in all hydrogel compositions secreted numerous ECM proteins. Immunostaining for fibronectin, which strongly incorporated the SILAC label, identified it pericellularly, where it co-localized with punctate α_5_ integrin (Fig. 4b). This suggests that integrin-mediated interactions could have taken place and that differentiation here could have been driven, at least in part, by actin-mediated cytoskeletal tension mediated via the secreted pericellular matrix, despite cells’ round morphologies (Fig. 4c). However, recent reports have also suggested that microtubules may also have a role in mechanotransduction-mediated differentiation in the absence of cell spreading^9^ via the microtubule-associated protein DCAMKL1, which antagonizes RUNX2 transcriptional activity^39^. Treating cells with paclitaxel, an inhibitor of microtubule depolymerization, eliminated composition-dependent differences in gene expression, precluding upregulation of *RUNX2* and *BGLAP* in 1:0.75 hydrogels and abolishing upregulation of *PPARγ* and *C/EBPα* in 1:0.375 and 1:3 hydrogels (Fig. 4d and Supplementary Fig. 18). As staining encapsulated hMSC for tubulin revealed a well-defined network (Fig. 4e), these observations suggest that the microtubule network may have a role in regulating gene expression under these conditions.

## Discussion

In summary, hMSC within S-HA-PEGDA hydrogels quickly modify the composition and physical characteristics of their pericellular space and interact with it to direct their fate. Here, we found that pericellular matrix deposition drove adipogenesis in hydrogels that were inefficiently cross-linked (1:0.375/1:3), but matrix degradation promoted osteogenesis when hydrogel cross-linking was more efficient (1:0.75). Cross-linking efficiency will impact numerous hydrogel properties, including mesh size, stiffness, and the susceptibility of the hydrogel to enzymatic degradation and matrix deposition. We were not able to identify which specific property mediated our observations here, but all remain possibilities. Our proteomic analysis identified proteins secreted by hMSC that were retained in the hydrogels, but did not identify which or if specific proteins directed cellular responses. As many proteins often act in concert in vivo to direct cell behaviours, it is likely that numerous proteins were similarly involved here. In our experiments, we kept the concentration of HA constant in all hydrogel compositions, but we cannot rule out its own biological influence, as HA can modulate cellular mechanotransduction^40^. Moreover, whereas others have linked osteogenesis to cell spreading and traction^5^, our observations suggest that cell-mediated degradation in the absence of cell spreading may be sufficient, confirming previous reports^4,19,41^. Our observations of cell-mediated modifications of pericellular matrix may explain conflicting observations in different hydrogel systems, despite similar stiffnesses and ligand presentation, as hydrogel properties per se may not always directly regulate hMSC, but rather impact the formation of pericellular matrix, which in turn influences fate. Indeed, our data suggest that hMSC participate in a reciprocal interplay between the 3D milieu they are presented with and their own secreted pericellular matrix and this combination of interactions directs fate.

## Methods

### Hydrogel preparation

Sodium hyaluronate (Lifecore Biomedical, USA, mean molecular weight 111 kDa) was thiolated as previously described^20,24,42,43^ to achieve a polymer degree of substitution between 30 and 40% of the d-glucuronic acid units. The degree of substitution was quantified by proton nuclear magnetic resonance (^1^H-NMR) spectroscopy (Supplementary Fig. 19) as previously described^20^, and confirmed using the Ellman procedure^44^. The resulting product was sterilized with 25 kGy gamma irradiation using a Gammacell 1000 (Best Theratronics Ltd., UK). As CD44 requires a hexasacchride to allow for binding^6^ and as higher molecular weight hyaluronic acid (HA) binds to more CD44 receptors^45^, we hypothesized that working in the molecular weight range of 100–150 kDa HA would allow cells access to multiple binding sites while still tolerating a reasonable degree of chemical modification^46^. And indeed, while others who have utilized more extensively modified HA report limited cell interactions^25^, blocking (Fig. 1b) and degradation (Supplementary Fig. 2) studies confirmed hMSC could interact with, and hyaluronidase could degrade HA with this degree of chemical modification.

Cylindrical hydrogels (100 μL volume) were prepared in polytetrafluoroethylene molds (6 mm diameter) by combining a constant amount of S-HA (8 mg mL^−1^) and varying the concentration of PEGDA (ESI-BIO, USA, 3400 Da, 1.5–48 mg mL^−1^), as outlined in Supplementary Table 1. S-HA and PEGDA were both solubilized in phosphate buffered saline (PBS, without calcium and magnesium). The solution was then pipetted into molds and allowed to crosslink at 37 °C in a humidified atmosphere of 5% CO_2_/95% air for 2 h prior to the addition of basal culture medium (alpha modified Eagle’s medium, αMEM, no nucleosides) with 10% Fetal Bovine Serum (FBS, Gibco, UK) and 1% (v/v) antibiotic-antimycotic solution (Sigma) and removal of the molds.

### Mechanical characterization of hydrogels by AFM

Spherical glass beads (diameter 55–75 μm; Polysciences, Inc, DE) were mounted onto tipless triangular silicon nitride cantilevers (spring constant (*K*) ≈ 0.06–0.24 N m^−1^; Bruker AXS SAS, FR) using UV-cross-linked Loctite super glue (Hemel Hempstead, UK). The diameter of each microsphere was then measured on a Leica MZ FL III fluorescence stereomicroscope with a Leica DFC300 FX digital colour camera using Leica FireCam software (Leica Microsystems, CH) using a standard scale. Prior to any sample measurements, the deflection sensitivity of the AFM photodiode was calibrated by performing a single force-distance curve on a glass slide following standard protocols. Cantilevers were then calibrated using the thermal method^47^ in air and repeated up to three times to confirm the value of the spring constant. Hydrogels ~3 mm in thickness (to minimize the stiffness contribution from the underlying support^48^) were immobilized in 60 mm diameter tissue culture dishes (TPP, CH) by gluing coverslips to the plate at the edges of the hydrogel. In this setup, the coverslips act as clamps on the edge of the hydrogels, avoiding the need of gluing the hydrogel to the petridish. The mounting of the hydrogels was sufficient to hold them in place without exerting compressive load in the area probed by AFM. The hydrogels were kept hydrated (PBS) at room temperature (RT) at all times during the preparation, mounting, and force-distance measurements to ensure that their physical properties were not altered. Unfixed or otherwise manipulated hydrogels, with or without live cells, were taken directly from the cell culture incubator, immersed in ~4.5 mL of PBS at RT (measurements both in the presence and absence of live cells were completed within 1 h with a resting time of 10 min after mounting for thermal equilibrium stabilization) and measurements were carried out on a JPK Nanowizard I AFM with J Unicam using JPK SPM software 2.3 01/2006 (JPK Instruments AG, DE). Force measurements were made at randomly selected locations across the hydrogel surface (at least 10 locations and 10 force curves per location on at least two technical replicate hydrogels per biological replicate). Randomly selected areas were always at least 100 µm from each other. The total number of force curves that were used for each analysis are given in Supplementary Table 2. Indentations were carried out with a relative setpoint force of 30 nN and a loading rate of 30 μm s^−1^. *E* was determined with JPK SPM software using the Oliver–Pharr model^49^ for a spherical tip, as previously described^48^. This analysis is based on an elastic–plastic contact mechanical model. As for other hydrated biological samples, we assumed that volume was conserved and assigned a Poisson’s ratio of 0.5.

Histograms were plotted with identical bin sizes for each hydrogel composition and *E* was then determined by fitting a Gaussian distribution to the histogram (only when tolerance criteria were 100% satisfied in OriginPro 8 SR0 software) and identifying the centre of the peak. Standard error was determined as the half width of the Gaussian peak, as previously described^50^.

### hMSC isolation, culture, and characterization

Human samples used in this research project were obtained from the Imperial College Healthcare Tissue Bank (ICHTB, HTA license 12275). ICHTB is supported by the National Institute for Health Research (NIHR) Biomedical Research Centre based at Imperial College Healthcare NHS Trust and Imperial College London. ICHTB is approved by the UK National Research Ethics Service to release human material for research (12/WA/0196), and the samples for this project were issued from sub-collection R16052. Clinical-grade, bone marrow-derived hMSC were generated from bone marrow (BM) aspirates collected from the iliac crest of healthy paediatric donors with informed consent. BM aspirate was collected in 10 mL tubes and cells were plated the same day in CellSTACK^®^ (Corning, UK) culture chambers at 10–25 × 10^6^ per 636 cm^2^ after establishing the total number of nucleated cells on a Sysmex SE full blood count analyzer. Cells were cultured in αMEM supplemented with 5% human platelet lysate (Stemulate, Cook Medical, USA) and cultured under standard conditions. When cell confluence of 90–100% was achieved (10–14 days), cells were detached with recombinant trypsin (Roche, DE) and reseeded at 5000 cells per cm^2^. Cells were then routinely expanded in αMEM supplemented with 10% (v/v) FBS (using 0.05% Trypsin-EDTA, Thermo Fisher Scientific) and used prior to passage 7. Cells were immunophenotyped with a panel of labeled mouse anti-human antibodies and found to express markers CD90, CD105, CD73, and not express haematopoietic markers CD34 and CD45^51^. Immunophenotyping was performed at passages 0, 1, 4, and 7. hMSC were tested and devoid of mycoplasma contamination before being used in experiments.

### hMSC encapsulation in hydrogels

To encapsulate cells, hMSC were trysinized at ~80% confluency and pelleted. Cells were resuspended in αMEM to constitute 8% of total final hydrogel volume, mixed with hydrogel components and pipetted into molds placed within 48-well suspension plates. Hydrogels were allowed to cross-link under standard culture conditions for 2 h before molds were removed and 1 mL of basal culture medium supplemented with 1% (v/v) antibiotic-antimycotic solution was added. Media were exchanged every 3–4 days.

### SILAC media, amino acids, and dialysed serum for proteomics

SILAC αMEM, no nucleosides, no lipoic acid was made up in-house under sterile conditions. Heavy SILAC medium was prepared with 0.1 mg mL^−1^ heavy isotopically labeled lysine (CNLM-291-H, Cambridge Isotope Laboratories, Inc) and heavy isotopically labeled arginine (CNLM-539-H, Cambridge Isotope Laboratories, Inc). Unlabeled Light SILAC media was prepared with 0.1 mg mL^−1^ l-arginine (A8094, Sigma) and l-lysine (L8662, Sigma). FBS was extensively dialyzed with PBS using Amicon 3K MWCO units (Millipore, UK). Viability of hMSC seeded on tissue culture plastic (TCP) or encapsulated within hydrogels was evaluated using SILAC and control culture media as described in the Supplementary Methods (Supplementary Fig. 20).

### Cell-laden hydrogel preparation for proteomic analysis

hMSC were extensively washed with PBS and then trypsinized. Trypsin was neutralized with appropriate SILAC αMEM, no nucleosides, no lipoic acid supplemented with 10% dialyzed FBS. Some cell suspensions were extensively washed with Heavy SILAC αMEM (without serum). Cell-laden hydrogels (100 μL, *n* = 3) were then prepared and cultured for 72 h under standard conditions with Heavy SILAC supplemented with 10% dialyzed FBS and 1% antibiotic-antimycotic solution. To generate time-course data, cell suspensions were treated identically with the exception that cells were washed and cultured with Light SILAC medium. After 24 or 48 h, Light SILAC medium was replaced by heavy SILAC medium and hydrogels cultured for 72 h in total. Samples were decellularized with 0.5% Triton X-100 in PBS supplemented with 20 mM ammonium hydroxide (both Sigma) by gentle agitation for 90 min at RT^52^. Samples were then extensively washed with PBS and stored in PBS supplemented with 1% antibiotic-antimycotic solution at 4 °C. The efficiency of hydrogel decellularization was confirmed by fluorescence microscopy by staining with 1 μg mL^−1^ 4′,6-diamidino-2-phenylindole (DAPI, #D3571, Molecular Probes), and 1:100 Phalloidin-Tetramethylrhodamine B isothiocyanate (TRITC)^53^ from *Amanita phalloides* (#P1951, Sigma).

### In-hydrogel digestion for proteomic analysis

Each hydrogel was incubated for 10 min at RT with 100 µL acetonitrile (Optima, LC/MS Grade, Fisher Chemical), which caused them to shrink and whiten in colour. Each hydrogel was then transferred to a 2 mL tube and the acetonitrile was removed. Lyophilized trypsin (Sequencing Grade Modified Trypsin, Promega) was made up to 100 ng µL^−1^ in 50 mM acetic acid, then diluted ten-fold in 100 mM ammonium bicarbonate to give a 10 ng µL^−1^ working solution. 50 ng trypsin was added to the hydrogel and allowed to absorb into the hydrogel for 30 min at RT. 100 µL 100 mM ammonium bicarbonate was then added and the hydrogel was incubated overnight at 37 °C on a bench top mixer set to mix at 800 rpm. The tube was centrifuged (425 rcf, 3 min, 25 °C) and the supernatant (containing peptides) was transferred to a new 2 mL tube. 30 µL acetonitrile was added to the hydrogel and incubated for 5 min at RT. The tube was then centrifuged (425 rcf, 3 min, 25 °C) again and the supernatant was combined with the previous supernatant creating the final peptide sample. The sample was vacuum centrifuged to dryness for 30 min then a C18 reversed phase clean-up was performed using in-house prepared StageTips^54^. Peptides were finally eluted from the StageTip with 80% acetonitrile/5% trifluoroacetic acid (TFA) and vacuum centrifuged to dryness.

### Mass spectrometry (MS) analysis

Dried peptides were re-suspended in 15 µL 0.1% TFA, sonicated for 10 min and centrifuged (20,817 rcf, 3 min, 25 °C). 5 µL of supernatant was transferred to a glass autosampler vial containing 45 µL of 0.1% TFA to give a 1:10 dilution. This was determined as an appropriate concentration based on preliminary experiments. 10 µL sample was analyzed in technical duplicate using a ThermoFisher Scientific QExactive mass spectrometer coupled to an UltiMate 3000 HPLC system for on-line liquid chromatographic separation. The sample was initially loaded onto a C18 trap column (ThermoFisher Scientific Acclaim PepMap 100; 5 mm length, 300 µm inner diameter) then transferred onto a C18 reversed phase column (ThermoFisher Scientific Acclaim PepMap 100; 50 cm length, 75 µm inner diameter). Peptides were eluted with a linear gradient of 5–40% buffer B (80% acetonitrile, 0.1% formic acid, 5% DMSO) at a flow rate of 250 nL min^−1^ over 35 min.

Higher energy collisional dissociation was selected as the activation method. Singly-charged and unknown charge state precursor ions were not analyzed. Full MS spectra were acquired in the orbitrap (*m*/*z* 300–1800; resolution 70k; AGC target value 1E6) with the MS/MS spectra of the ten most abundant precursors from the preceding MS survey scan then acquired (resolution 17.5k, AGC target value 1E5; normalized collision energy 28 eV; minimum AGC target 1E2). Selected precursors were dynamically excluded for 15 s.

### Proteomic data analysis

Acquired raw data were analyzed with MaxQuant software (version 1.3.0.5)^55^. MaxQuant was set up to analyze SILAC samples by selecting a multiplicity of 2 and heavy labels Arg10 and Lys8. MS/MS spectra were searched against a UniProtKB human protein database (downloaded May 2017; 159,743 entries) using the Andromeda search engine. Common protein contaminants were included in the search. Trypsin enzyme was selected (C-terminal cleavage of arginine and lysine residues) and a maximum of two missed cleavages was permitted. Carbamidomethylation of cysteine was set as a fixed modification; N-terminal protein acetylation and methionine oxidation were set as variable modifications. All other MaxQuant parameters were maintained at default including a peptide false discovery rate of 0.01.

The resulting protein Groups.txt file was opened in Microsoft Office Excel 2016. Percentage heavy label incorporation values were created for each protein using the formula “(*R*/(1 + *R*)) × 100%”, where *R* is the ratio of the heavy and light peptide signal as calculated by MaxQuant. The file was saved as a txt file and then opened in Perseus (version 1.4.0.2)^56^. For each sample, a minimum ratio *H*/*L* count threshold of 3 for both technical duplicates was set. The ratio *H*/*L* count refers to the number of peptide quantification events used for protein quantification. Technical replicates were plotted using the scatterplot function. Gene ontology annotation was performed to identify ECM proteins.

### Visualization of protein production

Cell-laden hydrogels (*n* = 3) were prepared as described above in high glucose DMEM without glutamine, methionine, or cystine (#21013024, Life Technologies) supplemented with 4 mM l-glutamine, 0.201 mM l-cystine (#C7602), 1 mM sodium pyruvate, 50 μg mL^−1^ ascorbate 2-phosphate, and 40 μg mL^−1^ proline (all form Sigma). Cell-laden hydrogels were cultured for 72 h in medium supplemented with 1.25 mg mL^−1^ bovine serum albumin (Sigma), 0.1% insulin-transferrin-selenium (Thermo Fisher Scientific), 1% (v/v) antibiotic-antimycotic solution and with either 0.1 mM l-methionine (#M5308, Sigma; control media) or 0.1 mM l-homopropargylglycine (HPG, #C10186, Molecular Probes; labeling media). HPG was then identified using a click-IT HPG Alexa Fluor 488 protein synthesis assay kit (#C10428, Molecular Probes), following the manufacturer’s instructions at RT. Briefly, samples were fixed in 4% (w/v) paraformaldehyde (PFA) in PBS for 20 min, rinsed twice with PBS, and incubated for 1 h with the labeling solution. Samples were then washed for 5 min in reaction rinse buffer, and then once with PBS. Nuclei were labeled with NuclearMask^TM^ in PBS for 15 min, and samples were washed thrice with PBS before imaging. Controls were treated similarly to labeled samples except the labeling media was replaced with control media, which did not contain HPG (Supplementary Fig. 21). Samples were coverslipped using PBS and imaged as intact hydrogels on a Leica DM16000 confocal laser scanning microscope (Leica Microsystems, CH). Detector gains were set to be constant between samples to facilitate comparison. Differential interference contrast (DIC) imaging was used to delineate the cell membrane. *Z*-series with 2 µm *Z*-spacing were obtained in randomly selected fields using sequential acquisition and Kalman filter mode with a ×40 oil objective with a numerical aperture of 1.25, and 2048 × 2048 pixel size. Images show 3D projections obtained using the Nikon’s NIS-Elements AR 4.51.00 software and insets correspond to max projections of 5 central *Z*-slices of 2 μm obtained using Image J. Only in cells which we were able to capture the entire cell and its secreted pericellular matrix, 2 central Z-slices were selected, and 20 random lines were generated by drawing radii extending from the cell membrane (cell membrane identified by DIC, scheme and example analyses are shown in Supplementary Fig. 21). The fluorescence intensity of the labeled secreted protein as function of distance from the cell membrane was then measured in radial profile plots using the Image J plugin Multi plot. Each value of intensity (arbitrary units) corresponds to a single pixel at the indicated distance from the cell membrane. A total of 40 measurements were performed per cell, and 30 cells were analyzed per condition (total of 1200 plots of intensity vs. distance from the cell membrane). Values reported are the mean ± s.e.m. The effect of the hyaluronidase inhibitor Vcpal (100 μM) on hMSC protein secretion was determined identically, but cell-laden hydrogels (*n* = 3) were treated with the inhibitor (Vcpal+) for 72 h prior to analysis.

### Gene expression analyses of hMSC within hydrogels

Hydrogels were snap frozen in liquid nitrogen, stored at −80 °C, and then homogenized in Lysing Matrix H tubes (MP Biomedicals, USA) on a FastPrep^®^−24 (MP Biomedicals, 4.5 m s^−1^ for 2 cycles of 10 s). RNA was isolated using an RNeasy Mini Kit according to the manufacturer’s instructions using QIAshredder and the RNase-Free DNase Set (all from Qiagen, NL). RNA was eluted in 30 μL of UltraPure™ DEPC-Treated Water (Invitrogen) and reverse transcribed into cDNA in two steps. First 10 μL of RNA, 2 μL 500 μg mL^−1^ random primers (Promega), and 2 μL of RNase-free water were incubated at 70 °C for 5 min. Next, the reaction mixture was mixed with 1.25 μL 1 mM PCR Nucleotide Mix (Promega), 5 μL M-MLV reverse transcriptase 5× buffer, 1 μL M-MLV 200 U μL^−1^ reverse transcriptase (Promega), and 3.75 μL RNase free water and incubated for 1 h at 42 °C. cDNA was stored at −20 °C. Quantitative polymerase chain reaction (qPCR) was performed on a Bio-Rad CFX384 Touch^TM^ real-time PCR detection system using 384-Well PCR Plates (all from Bio-Rad Laboratories Ltd., UK). Reaction mixtures were prepared with 5 μL 2× Brilliant III SYBR Green qPCR Master Mix (Agilent, USA), 3 μL of primer mix (forward and reverse), 1.5 μL RNase-free water and 0.5 μL of cDNA. A three-step cycle was employed: (1) denaturation at 95 °C for 3 min; (2) annealing/extension at 58 °C for 10 s; and (3) melting from 65 °C to 95 °C at 0.5 °C per step for 5 s.

Reference genes (RG) were chosen after testing their stability over time in hMSC encapsulated in hydrogels as RG stability is known to vary when cells are combined with materials^57^, or in 2D vs. 3D culture^58^. Briefly, serial dilutions of samples pooled from different conditions (time in culture, PEGDA concentration, and cell density) were ranked according to repeatability of the gene expression differences. Geometric means calculated in RefFinder which combines Comparative ΔCT^58^, BestKeeper^59^, Normfinder^60^, and geNorm^61^ indicated the most stable RG (Supplementary Table 4). Primers (Integrated DNA Technologies, UK) were designed with Primerbank^57^ and Primer Blast (Supplementary Table 5). Samples (*n* ≥ 4) were run in triplicate and water was used in place of cDNA as a control. The ΔΔCT method^62^ was used to quantify fold changes in the expression for each gene of interest (GOI) and normalized to the expression of undifferentiated hMSC (day 0), using *RPL13a* and *EEF1A1* as the RG: fold change in expression = 2^−^^ΔΔCq^, ΔΔCq = [(Cq_GOI,t(x)_ – Cq_RG,t(x)_) – (Cq_GOI,t(0)_ – Cq_RG,t(0)_)]. Ratio of expression of *PPARγ* to *RUNX2* was calculated using the fold change in expression of *PPARγ* normalized to the average fold change in expression of *RUNX2*. Data shown are means + s.d. of the biological replicates.

### Assessing hMSC terminal differentiation in hydrogels

Cell-laden hydrogels (5 × 10^5^ cells mL^−1^, *n* = 3) were incubated for 14 days in co-induction medium (adipogenic/osteogenic co-inductive medium mixed in a 1:1 ratio) that was exchanged every 3–4 days. Adipogenic inductive medium was prepared with αMEM supplemented with 10 % (v/v) FBS, 1% (v/v) antibiotic-antimycotic solution, 1 μM dexamethasone, 0.2 mM indometacin, 10 μg ml^−1^ insulin, 0.5 mM 3-isobutyl-1-methilxantin and 3.5 g mL^−1^ glucose (all from Sigma). Osteogenic medium was prepared with αMEM supplemented with 10 % (v/v) FBS, 1% (v/v) antibiotic-antimycotic solution, 0.2 mM ascorbic acid, 10 mM β-glycerophosphate, 10 nM dexamethasone and 3.5 g mL^−1^ glucose (all from Sigma). Additional cell-laden hydrogels (*n* = 3) were also treated with Exo-1 (75 μM) or Vcpal (100 μM) for 14 days in co-induction medium.

After culture, hydrogels were fixed in 4% (v/v) PFA in PBS for 20 min. After washing in PBS, samples were treated with 0.1% (v/v) Triton-X-100 and 0.1% (v/v) Tween-20 for 30 min at RT. Alkaline phosphatase (ALP) activity and neutral lipid accumulation were visualized by ALP and Oil Red O (ORO) staining^35^, respectively, on whole mounted hydrogels. For ALP staining, samples were equilibrated in alkaline staining buffer (100 mM CaCl_2_, 100 mM Tris-HCl, 100 mM NaCl, 0.1% (v/v) Tween-20, 50 mM MgCl_2_, pH 8.2) for 20 min, and then incubated for 3 h at RT in the same buffer with the addition of 500 μg mL^−1^ naphthol and 500 μg mL^−1^ Fast Blue BB (all from Sigma). For ORO staining, samples were equilibrated for 20 min in PBS with 100 mM CaCl_2_ and 0.1% Tween-20 and then stained for 2 h at RT with a solution of 600 μg mL^−1^ ORO in isopropyl alcohol (all from Sigma), both as previously described^4^. After two washes in the buffers mentioned above and two washes in PBS, nuclei were counterstained with 1 μg mL^−1^ DAPI for 30 min at RT. Samples were washed thrice with PBS, fixed a second time in 4% PFA with 100 mM CaCl_2_ and 0.1% (v/v) Tween-20, washed, and mounted in PBS. Color and fluorescence micrographs were acquired in randomly selected fields using an Axiovert 200 M Zeiss microscope and a QImaging Retiga R3 CCD camera using a ×20 objective. The fraction of hMSC stained with ALP or ORO were counted using DAPI counterstaining in more than 20 imaged fields per condition. In total, between 300–1200 cells were analyzed per condition.

### Inhibiting hyaluronidase activity and protein secretion

To determine appropriate inhibitors and doses that would either disrupt protein secretion or inhibit hyaluronidase activity, the following compounds were tested: L-ascorbyl palmitate (Vcpal; 10 mM stock solution in DMSO, Sigma) is a well-studied inhibitor of hyaluronidase activity;^32^ Golgicide A (35 mM stock solution in DMSO, Sigma) inhibits Golgi BFA resistance factor 1 (GBF1) function^63^, arresting secretion of soluble and membrane associated proteins; Brefeldin A (35 mM stock solution in DMSO, Sigma) disrupts protein translocation to the Golgi^64,65^; and Exo-1 (2-(4-Fluorobenzoylamino)-benzoic acid methyl ester, 35 mM stock solution in DMSO, Sigma) perturbs exocytosis as it modifies Golgi ARF1 GTPase activity and thus trafficking between the ER and Golgi^30^. Serial dilutions of all inhibitors were used to test hMSC viability on TCP and in 3D (Supplementary Methods). Inhibition of protein secretion was evaluated by immunostaining for fibronectin (Supplementary Methods, Supplementary Fig. 22 and Supplementary Table 6). The ability of Vcpal to inhibit hyaluronidase activity was evaluated as previously described^66^. Briefly, a 10 mM Vcpal stock solution was prepared in DMSO; hyaluronidase was prepared in PBS at 200 U mL^−1^ and both syringe filtered (0.22 µm). An enzyme/inhibitor solution was then prepared with 100 µM Vcpal per 100 U mL^−1^ hyluronidase and incubated for 1 h at 37 °C while shaking. The solution was centrifuged, and the supernatant retained. 2 mg mL^−1^ of S-HA and the enzyme/inhibitor supernatant were then mixed 1:1 and incubated at 37 °C while shaking for up to 48 h. Samples were loaded on 10% polyacrylamide gels for size separation (using PageRuler Plus Prestained Protein Ladder, 10 to 250 kDa, ThermoFisher) and images captured after staining with 0.005% Stains-All (E9379, Sigma) in a 50% ethanol/dH_2_O solution overnight at RT and destaining with 10% ethanol/dH_2_O. Exo-1 (75 μM) and Vcpal (100 μM) in basal culture medium were added 2 h after cell-laden hydrogels were formed. After 3 days under standard culture conditions, viability, gene expression analysis, and AFM measurements were performed.

### Blocking cell-ECM interactions

To block cell-ECM interactions, hMSC were pre-incubated for 45 min at 4 °C before encapsulation within hydrogels in basal culture medium (vehicle) or with 10 μg mL^−1^ mouse anti-CD44/H-CAM monoclonal IgG1 antibody [5F12] (#MA5-12394, Invitrogen, Thermo Fisher Scientific), or mouse IgG1, κ isotype control (#MA5-14453, Invitrogen, Thermo Fisher Scientific) both concentrated after 3 washes with PBS to remove sodium azide using Amicon 30 K MWCO units (Millipore, UK)^67,68^, or 300 μg mL^−1^ of RGD+ (Gly-Arg-Gly-Asp-Ser-Pro, GRGDSP, Bachem, CH), or 300 μg mL^−1^ RGD- (Gly-Arg-Ala-Asp-Ser-Pro, GRADSP, Bachem)^69,70^ peptides (*n* ≥ 5). After encapsulation, media containing the antibody or peptides (at the same concentrations) were added to appropriate wells. Data for viability and gene expression are presented as means + s.d. of the biological replicates.

### Inhibition of RhoA/ROCK and microtubule depolymerization

To block Rho-associated, coiled-coil containing protein kinase (ROCK) activity, hMSC-laden hydrogels (*n* ≥ 5) were cultured in basal medium (vehicle) or treated with 10 μM Y-27632 (Y, [(1)-(*R*)-trans-4-(1-aminoethyl)-*N*-(4-pyridyl)cyclohexanecarboxamide dihydrochloride], Merck) for 72 h. To inhibit microtubule disassembly, hMSC-laden hydrogels (*n* ≥ 5) were treated with 50 nM paclitaxel (PTX, #ab120143, Abcam) for 72 h. Vehicle controls were cultured in basal culture medium. Data for viability and gene expression are presented as means + s.d. of the biological replicates.

### Statistical analyses

For all in vitro experiments, samples were prepared, treated, processed and analyzed in random order. No statistical method was used to pre-determine sample size, and experiments were performed without blinding. Measurements of *E* (after removal of outliers with ROUT test *Q* = 1%) are shown as histograms (percentage) and box plots expressing the median (central line), first and third quartiles (bounds of box), and the highest and lowest values (whiskers). Flow cytometry results are shown as medians with s.e.m. All other results are shown as means and s.d. or s.e.m. Statistical analyses were carried out using a non-parametric Kruskal–Wallis test followed by Dunn’s multiple comparison test for multiple comparisons or a Mann–Whitney test (two-tailed) for comparisons between two groups. Statistical analyses for ORO or ALP quantification were performed by Fisher’s exact test (two-sided). Significant differences in the distributions of stiffness values between acellular and cell-laden hydrogels, or treated with Exo-1 or Vcpal were evaluated using a Mantel–Haenszel linear-by-linear association chi-squared (*χ*^2^) test for trend. Power was evaluated by determining Goodman and Kruskal’s gamma (*γ*) and standardized residuals were used to identify the most significant intervals that contributed to differences between histograms, all three using IBM^®^ SPSS^®^ statistics version V23. All other statistical analyses were carried out using GraphPad Prism version 7 for Windows (GraphPad Software, USA). *p* values are indicated in figure captions or can be found in Supplementary Tables 2 and 3.

## Electronic supplementary material

Supplementary Information

Description of Additional Supplementary Files

Supplementary Data 1

## Data Availability

The full mass spectrometry proteomics data obtained in this study have been deposited with the ProteomeXchange Consortium via the PRIDE partner repository with the dataset identifier PXD009500. The authors declare that all data supporting the findings of this study are available within the article and its supplementary information files or from the corresponding author upon reasonable request.
