# Author Correction: Bi-directional cell-pericellular matrix interactions direct stem cell fate

**DOI:** 10.1038/s41467-018-07398-1

**Published:** 2018-11-14

**Authors:** Silvia A. Ferreira, Meghna S. Motwani, Peter A. Faull, Alexis J. Seymour, Tracy T. L. Yu, Marjan Enayati, Dheraj K. Taheem, Christoph Salzlechner, Tabasom Haghighi, Ewa M. Kania, Oommen P. Oommen, Tarek Ahmed, Sandra Loaiza, Katarzyna Parzych, Francesco Dazzi, Oommen P. Varghese, Frederic Festy, Agamemnon E. Grigoriadis, Holger W. Auner, Ambrosius P. Snijders, Laurent Bozec, Eileen Gentleman

**Affiliations:** 1 Centre for Craniofacial and Regenerative Biology, King’s College London, London SE1 9RT, UK; 2 Protein Analysis and Proteomics Platform, The Francis Crick Institute, London NW1 1AT, UK; 3 Bioengineering and Nanomedicine Lab, Faculty of Biomedical Sciences and Engineering, Tampere University of Technology and BioMediTech Institute, 33720 Tampere, Finland; 4 Biomaterials and Tissue Engineering, Eastman Dental Institute, University College London, London WC1X 8LD, UK; 5 Cancer Cell Protein Metabolism Group, Department of Medicine, Imperial College London, London W12 0NN, UK; 6 Department of Haemato-Oncology, Rayne Institute, King’s College London, London SE5 9NU, UK; 7 Department of Chemistry, Ångström Laboratory, Science for Life Laboratory, Uppsala University, SE-75121 Uppsala, Sweden; 8 Tissue Engineering and Biophotonics, King’s College London, London SE1 9RT, UK; 9 Faculty of Dentistry, University of Toronto, 124 Edward Street, Toronto, Toronto ON M5G 1G6, Canada

Correction to: *Nature Communications* 10.1038/s41467-018-06183-4; published online 03 October 2018.

In the original version of this Article the dataset identifier in the Data Availability statement was incorrect. The correct dataset identifier is PXD009500. This has been corrected in the HTML and PDF versions of this Article.

