# Author Correction: Bi-directional cell-pericellular matrix interactions direct stem cell fate

**DOI:** 10.1038/s41467-018-07843-1

**Published:** 2018-12-18

**Authors:** Silvia A. Ferreira, Meghna S. Motwani, Peter A. Faull, Alexis J. Seymour, Tracy T. L. Yu, Marjan Enayati, Dheraj K. Taheem, Christoph Salzlechner, Tabasom Haghighi, Ewa M. Kania, Oommenp P. Oommen, Tarek Ahmed, Sandra Loaiza, Katarzyna Parzych, Francesco Dazzi, Oommen P. Varghese, Frederic Festy, Agamemnon E. Grigoriadis, Holger W. Auner, Ambrosius P. Snijders, Laurent Bozec, Eileen Gentleman

**Affiliations:** 1 Centre for Craniofacial and Regenerative Biology, King’s College London, London, SE1 9RT UK; 2 Protein Analysis and Proteomics Platform, The Francis Crick Institute, London, NW1 1AT UK; 3 Ludwig Boltzmann Cluster for Cardiovascular Research at the Center for Biomedical Research, Medical University of Vienna, Vienna, Austria; 4 Bioengineering and Nanomedicine Lab, Faculty of Biomedical Sciences and Engineering, Tampere University of Technology and BioMediTech Institute, 33720 Tampere, Finland; 5 Biomaterials and Tissue Engineering, Eastman Dental Institute, University College London, London, WC1X 8LD UK; 6 Cancer Cell Protein Metabolism Group, Department of Medicine, Imperial College London, London, W12 0NN UK; 7 Department of Haemato-Oncology, Rayne Institute, King’s College London, London, SE5 9NU UK; 8 Department of Chemistry, Ångström Laboratory, Science for Life Laboratory, Uppsala University, SE-75121 Uppsala, Sweden; 9 Tissue Engineering and Biophotonics, King’s College London, London, SE1 9RT UK; 10 Faculty of Dentistry, University of Toronto, 124 Edward Street, Toronto, Toronto ON, M5G 1G6 Canada

**Keywords:** Stem-cell differentiation, Biomaterials - cells

Correction to: *Nature Communications* 10.1038/s41467-018-06183-4, published online: 03 Oct 2018

The original version of this Article contained an error in the author affiliations.

The affiliation of Marjan Enayati with ‘Ludwig Boltzmann Cluster for Cardiovascular Research at the Center for Biomedical Research, Medical University of Vienna, Austria' was inadvertently omitted.

This has now been corrected in both the PDF and HTML versions of the Article.

