# Supplementary material for: Bi-directional cell-pericellular matrix interactions direct stem cell fate
Source: Nat Commun. 2018 Oct 3;9:4049. doi: 10.1038/s41467-018-06183-4 (PMC6170409; doi:10.1038/s41467-018-06183-4)
Supplement: Supplementary file 1 — Supplementary Information [file 41467_2018_6183_MOESM1_ESM.pdf]

Supplementary Information

Ferreira, S.A. *et al*. "Bi-directional cell-pericellular matrix interactions direct stem cell fate"

Supplementary Methods

Hydrogel wet weight changes in basal culture medium

To determine bulk changes in the wet weight of acellular hydrogels over time in basal culture medium as an indicator of swelling behaviour, hydrogels (100 µL, *n* = 6) were prepared in pre-weighed vials and allowed to crosslink for 2 h as described in the Methods. After crosslinking, hydrogels were weighed to establish X_0, and basal culture medium was added to each vial. Hydrogels were maintained under standard culture conditions for 28 days. At selected time points, the supernatant was removed and hydrogels were weighed to determine X_t. Normalized changes in wet weight were determined by calculating X_t-X_0/X_0. In cell-laden hydrogels (100 µL, *n* ≥ 3), bulk hydrogel dimensions were assessed by measuring cross-sectional area[1]. After culture for 3 days under standard conditions, culture medium was removed and the bottom of the tissue culture plate was scanned (Epson Stylus SX130; Epson, UK). The cross-sectional area of hydrogels was then calculated using Image J. Pixels were converted to units of measurement using the known size of the wells. Data are shown as mean + s.d. of the biological replicates.

Hydrogel degradation

Hydrogels (100 µL, *n* ≥ 3) were prepared as described in the Methods. Degradation was determined by measuring the release of the uronic acid D-glucoronic acid in the presence of hyaluronidase by carbazole assay, as previously described[2]. Briefly, hydrogels were incubated in 2 mL 100 U mL^{-1} hyaluronidase (HAse, 60 kDa) from bovine testes (type I-S, 587 U mg^{-1}, Sigma) in PBS. PBS alone was used as a control. At selected time points, 40 µL of supernatant was removed and heated to 99 °C for 15 min. After cooling to 4 °C, 200 µL of 0.025 M sodium tetraborate·10 H_2O in concentrated sulfuric acid was then added to the samples, the solution heated to 99 °C for 15 min and then cooled to 4 °C. 8 µL of 0.125% (w/v) carbazole (Sigma) in absolute ethanol was then added and samples were briefly vortexed. After heating again to 99 °C for 15 min and cooling to 4 °C, the absorbance of the solution was measured at 495 nm at on colorimetric plate reader. The amount of D-

glucoronic acid in samples was then determined using solutions of known concentrations of D-glucoronic acid sodium salt. Data shown are normalized means ± s.d. of the biological replicates.

Hydrogel diffusivity

Hydrogels (150 µL, $n = 4$) were prepared in Lab-Tek II 8-well chamber slides (Thermo Fisher Scientific). 200 µL of a 1.4 mg mL^{-1} fluorescein isothiocyanate (FITC)-labeled dextran (70 kDa, 500 kDa or 2000 kDa, Sigma) solution in PBS was then added to each well. Hydrogels were allowed to incubate with the probe for 4 h under standard culture conditions prior to experiments. All experiments (12 measurements for each replicate) were performed at 37 °C on a Leica TCS SP5 confocal microscope with a dry 20× objective and pinhole of 2.00 airy units. A 25 µm diameter circle was selected for the bleaching area and was monitored at 496-603 nm during 10 pre-bleach scans (1.703 s total) at 5% laser intensity. The selected area was then bleached for 100 iterations (18.9 s total) at 100% laser intensity. Fluorescence recovery was monitored at 5% laser intensity to collect 300 further scans using a bi-directional scan speed of 1400 Hz. All scans created 512×512 pixel images and were taken at a 6× zoom. Image analysis to obtain measurements of half-life ($t_{1/2}$) were performed using the LAS AF application wizard software (Leica) and the diffusion coefficient (D) was calculated with the Soumpasis equation[3]: D (µm^2 s^{-1}) = $0.224\ r^2 / t_{1/2}$, where r is the radius of the cylindrical bleached spot. To compare relative diffusivities, the D of each hydrogel composition is plotted relative to the mean diffusion coefficient measured for PBS (D_0). Data shown are means ± s.d. of the biological replicates.

Evaluation of cell surface molecule thiols

hMSC ($n \geq 8$; 250,000 cells per well in 6-well plates) were extensively washed with PBS, trypsinized and the cell pellet was washed twice with cold PBS. Cells were incubated for 15 min on ice with 5 µM Alexa Fluor 488 C5 Maleimide (ALM, Thermo Fisher Scientific, Life Technologies) in PBS. Alexa Fluor 488 C5 Maleimide binds to thiol groups on cell surface

molecules (csm-SH)[4]. For negative controls, the cell suspension was treated for 15 min with 5 μM N-ethylmaleimide (NEM, Sigma) in cold PBS prior to incubation with ALM[4]. NEM is a membrane-permeable thiol-blocking agent that irreversibly replaces the hydrogen atom in SH groups[5]. For positive controls, attached cells were treated with 2 mM dithiothreitol (DTT, Sigma) in PBS at pH 8 for 15 min at 37 °C. The cell suspension was then incubated with ALM as described above. Between each step, cells were extensively washed with cold PBS and kept at 4 °C. For immunostaining analyses, cells were then stained with 1:1000 Hoechst 33342 (Thermo Scientific) and 8 μM ethidium homodimer-1 (Thermo Fisher Scientific) and imaged with Carl Zeiss Axiovert 200M inverted microscope with a Hamamatsu camera (Hamamatsu Photonics, Japan). For flow cytometry analyses, dead cells were identified with 500 ng mL^{-1} 4',6-diamidino-2-phenylindole (DAPI). Flow cytometric analysis was performed with a BD LSR FORTESSA™ cell analyzer using BD FACS Diva Software. Data shown are median (%) + s.e.m. of the biological replicates.

hMSC viability and metabolic activity within hydrogels

hMSC viability and metabolic activity when seeded on tissue culture plastic (TCP) was evaluated by conducting an alamarBlue® cell viability assay (Molecular Probes), according to the manufacturers' instructions. For the alamarBlue assay ($n \geq 3$), alamarBlue reagent 10% (v/v) in basal culture medium was added to each well and incubated under standard culture conditions for 4 h. 100 μL of supernatant was transferred to black 96 micro well-plates and fluorescence was read at 544 nm excitation and 590 nm emission in a FlexStation 3 multi-mode microplate reader (Molecular Devices). Data shown are means + s.d. of the biological replicates.

Cell viability when encapsulated within hydrogels ($n \geq 3$) was quantified using a Real Time-Glo™ MT cell viability assay (Promega, UK) according to the manufacturers' instructions. Luminescence was determined on a GLOMAX 96 well microplate luminometer (Promega). Data shown are means + s.d. of the biological replicates. In perturbation experiments (CD44,

RGD, Exo-1, Vcpal, Y-27632, PTX), results were normalized to the appropriate vehicle control.

Immunostaining of hMSC within hydrogels

At specified time points, hMSC-laden hydrogels were fixed in 4% (w/v) paraformaldehyde in PBS for 20 min. Cell-laden hydrogels were stained as intact bulk gels or embedded in a solution of 7.5% (w/v) gelatin, 15% (w/v) sucrose and 0.05% (v/v) sodium azide in PBS (all from Sigma) at 4 °C. Hydrogels were cryosectioned (10 µm) at -25 °C using a Bright Clinicut 60 cryostat (Bright Instrument Co Ltd., UK). Before staining, the gelatin solution was melted away in PBS at 37 °C. Hydrogel sections were permeabilized with 0.1% (v/v) Triton-X 100 in PBS (PBT) for 10 min and blocked for 1 h with 10% (v/v) sheep, goat or horse serum (as appropriate) in 0.15% (w/v) glycine and 0.2% (w/v) bovine serum albumin (Sigma) in PBT. Samples were stained overnight at 4 °C with primary antibodies or appropriate isotype controls (Supplementary Table 6 and Supplementary Fig. 22). In some cases, 1:100 Phalloidin-TRITC was also added. Slides stained for collagen were coverslipped using Fluoroshield™ mounting medium with DAPI (Sigma). Other samples were stained for 10 min with 1 µg mL^{-1} DAPI (#D3571, Molecular Probes), washed and coverslipped using ProLong Diamond Antifade Mountant (Molecular Probes).

Collagen imaging was carried out on a Nikon A1 confocal laser scanning microscope (Nikon). Other imaging was carried out on a Leica DM16000 confocal laser scanning microscope. Detector gains were set to be constant between samples to facilitate comparison. Z-series with 0.5 µm Z-spacing were obtained using sequential acquisition and Kalman filter mode, 63× glycerin objective with numerical aperture of 1.3, and 2,048 × 2,048 pixel size. The final point spread function calculated using Richard and Wolf 3D optical model was equal to 2. Colocalization between fibronectin (channel 1) and α_5 integrin (channel 2) was calculated using Image J plugin Coloc 2. Data presented are means ± s.d. of a total of 25 cells in 4 biological replicates and correspond to Manders' tM1 (above auto threshold on channel 2) and Manders' tM2 (above auto threshold on channel 1)[6]. Images for

collagen type II, PPARγ, C/EPBα, RUNX2, osteocalcin and isotype controls are max projections of 5 central Z-slices of 0.5 μm. The remaining images show 3D projections obtained using the Nikon's NIS-Elements AR 4.51.00 software and insets correspond to max projections of 5 central Z-slices of 0.5 μm obtained using Image J.

Degraded hydrogel fragments on hMSC differentiation

hMSC express hyaluronidases 1 and 2[7,8], which degrade HA to tetrasacchrides and ~20kDa fragments, respectively[9,10]. Therefore, acellular hydrogels (100 μL, *n* = 5) were cultured under standard cell culture conditions for 3 days with basal culture medium and then incubated for 24 h under the same conditions with a solution of hyaluronidase 1 from bovine (1000 U mL^{-1}, Sigma) and hyaluronidase 2 from sheep (1000 U mL^{-1}, Sigma) in αMEM (1 mL) to ensure complete degradation of the hydrogels and a heterogeneous mixture of different fragment sizes[11].

hMSC were seeded at 13,500 cells per cm^2 and allowed to attach for 24 h before experiments. Solutions containing hydrogel fragments and enzymes were inactivated at 100 °C for 5 min. Each mixture of fragments was then mixed 1:1 (v/v) with αMEM with 20% FBS (1 mL) and added to hMSC in individual wells of 12-well plates. Controls were created with a hyaluronidases solution only and cultured under the same conditions. Cells either treated with fragments or control solutions were then cultured under standard conditions for 3 days and cell viability was evaluated by alamarBlue® assay as described above. Cell lysates were collected using RLT buffer from the RNeasy Mini Kit and gene expression was carried out as described in the Methods. Data for viability and gene expression are presented as means + s.d. of the biological replicates.

Supplementary Tables

Supplementary Table 1: S-HA-PEGDA hydrogel compositions

Final concentration S-HA (mg mL^{-1})	Final concentration PEGDA (mg mL^{-1})	Weight ratio S-HA:PEGDA	Molar ratio thiol:acrylate*
8	1.5	1:0.1875	1:0.13
8	3	1:0.375	1:0.25
8	6	1:0.75	1:0.50
8	12	1:1.5	1:1.01
8	24	1:3	1:2.02
8	48	1:6	1:4.03

*The degree of thiol modification was determined by ^{1}H-NMR (mean modification of 35% of the disaccharide repeat units). The molecular weight of HA was assumed to be 400 g mol^{-1}.

Supplementary Table 2: Statistical analyses of distributions of *E* (histograms in Figure 2) of acellular and cell-laden hydrogels cultured under standard conditions or treated with either 75 µM Exo-1 or 100 µM Vcpal for 72 h

1:0.375

**acellular vs cell-laden**	**cell-laden vs Exo-1+**	**cell-laden vs Vcpal+**
$\chi^2 = 190.413$, $p < 0.001$<br>$\gamma = 0.828$, $p < 0.001$	$\chi^2 = 244.572$, $p < 0.001$<br>$\gamma = -1.000$, $p < 0.001$	$\chi^2 = 209.364$, $p < 0.001$<br>$\gamma = -0.840$, $p < 0.001$
SR: <2,000;<br>3,001-5,500	*SR*: 29-2,000;<br>3,001-5,500	*SR*: <500;<br>1,001-2,000;<br>3,001-5,500

$n_{\text{acellular}} = 294$ $n_{\text{cell-laden}} = 196$ $n_{\text{Exo-1+}} = 251$ $n_{\text{Vcpal+}} = 307$

1:0.75

**acellular vs cell-laden**	**cell-laden vs Exo-1+**	**cell-laden vs Vcpal+**
$\chi^2 = 159.296$, $p < 0.001$<br>$\gamma = -1.000$, $p < 0.001$	$\chi^2 = 135.150$, $p < 0.001$<br>$\gamma = -0.664$, $p < 0.001$	$\chi^2 = 112.916$, $p < 0.001$<br>$\gamma = 0.888$, $p < 0.001$
SR: <5,000;<br>7,501-20,000	*SR*: 250-500;<br>1,000-1,250;<br>1,500-1,750;<br>2,500-2,750;<br>3,000-3,250	*SR*: <2,500;<br>10,001-12,500;<br>17,501-25,000;<br>27,501-32,500

$n_{\text{acellular}} = 167$ $n_{\text{cell-laden}} = 168$ $n_{\text{Exo-1+}} = 313$ $n_{\text{Vcpal+}} = 216$

1:3

**acellular vs cell-laden**	**cell-laden vs Exo-1+**	**cell-laden vs Vcpal+**
$\chi^2 = 79.170$, $p < 0.001$<br>$\gamma = 0.448$, $p < 0.001$	$\chi^2 = 38.440$, $p < 0.001$<br>$\gamma = 0.09$, $p = 0.224$	$\chi^2 = 118.352$, $p < 0.001$<br>$\gamma = -1.000$, $p < 0.001$
SR: 501-1,000;<br>4,001-5,000;<br>5,501-6,500	*SR*: 1,001-1,500;<br>2501-3,500;<br>5,501-7,000;<br>8,501-9,000	*SR*: <1,000;<br>4001-5,000;<br>5,501-7,000;<br>8,501-9,000

$n_{\text{acellular}} = 175$ $n_{\text{cell-laden}} = 171$ $n_{\text{Exo-1+}} = 288$ $n_{\text{Vcpal+}} = 227$

A Mantel-Haenszel linear-by-linear association Chi-square test for trend (χ^2) (degrees of freedom = 1) was used to test if the distributions were significantly different from one another. A non-parametric Goodman and Kruskal's gamma (γ) test was then applied to measure of the strength of association that exists between any two comparisons. Low values for association indicate that two distributions are highly similar, while higher values are evidence of a stronger association and indicate that the distributions are different: none (0.00 ± 0.01), moderate association (± 0.10 – 0.29), strong association (± 0.30 – 0.99). Standardized residuals (*SR*), which highlight the most significant areas of the histograms that contributed to differences, coincided with the bi-modal distributions in cell-laden 1:0.375/1:3 hydrogels (compared to acellular controls), but identified the single shifted peak in 1:0.75 hydrogels.

Supplementary Table 3: Statistical analyses by Fisher's exact test (two-sided) of Oil Red O (ORO) and alkaline phosphatase (ALP) quantification of hMSC within S-HA-PEGDA hydrogels of different compositions after 14 days in culture in a bi-potential osteogenic/adipogenic medium and treated with either 75 µM Exo-1 or 100 µM Vcpal (Figure 3). Some significant changes in the fraction of hMSC stained positively for ORO and ALP in response to treatment with either Exo-1 or Vcpal likely reflect that in the absence of matrix-driven differentiation, the bi-potential chemical induction equally promotes osteogenesis and adipogenesis

ORO		
**1:0.375 vs 1:0.75**	**1:0.375 vs 1:3**	**1:0.75 vs 1:3**
$p < 0.001$	$p < 0.001$	$p < 0.001$

ALP		
**1:0.375 vs 1:0.75**	**1:0.375 vs 1:3**	**1:0.75 vs 1:3**
$p < 0.001$	$p < 0.001$	$p < 0.001$

ORO<br>Exo-1- vs Exo-1+		
**1:0.375**	**1:0.75**	**1:3**
$p < 0.001$	$p = 0.1729$	$p < 0.001$

ALP<br>Exo-1- vs Exo-1+		
**1:0.375**	**1:0.75**	**1:3**
$p = 0.0486$	$p < 0.001$	$p = 0.0025$

ORO<br>Vcpal- vs Vcpal+		
**1:0.375**	**1:0.75**	**1:3**
$p < 0.001$	$p = 0.0881$	$p < 0.001$

ALP<br>Vcpal- vs Vcpal+		
**1:0.375**	**1:0.75**	**1:3**
$p < 0.001$	$p < 0.001$	$p < 0.001$

Supplementary Table 4: Primers used to optimize reference genes for analyzing gene expression of hMSC encapsulated within S-HA-PEGDA hydrogels. After analyzing expression under various conditions of hydrogel culture, geometric means were calculated in RefFinder based on C_t values to determine each primer pair's stability. A low score indicates more stable expression

Gene	F/R	Sequence	Tm (°C)	Amplicon size	Primer Conc. (nM)	RefFinder Geometric mean of ranking values
RPL13A	F	GCC CTA CGA CAA GAA AAA GCG	60.14	117	150	1.57
	R	TAC TTC CAG CCA ACC TCG TGA	61.10			
ACTB	F	AAG GGA CTT CCT GTA ACA ATG CA	60.18	141	100	1.73
	R	CTG GGA ACG GTG AAG GTG ACA	62.25			
EEF1A1	F	GCT GAG CGT GAA CGT GGT AT	60.74	89	150	2.21
	R	CCT GGG GCA TCA ATG ATA GTC A	59.89			
HPRT1	F	GAA AAG GAC CCC ACG AAG TGT	60.48	89	100	3.98
	R	AGT CAA GGG CAT ATC CTA CAA CA	59.22			
GAPDH	F	CTC TGC TCC TCC TGT TCG ACA	61.49	204	100	4.23
	R	ACG ACC AAA TCC GTT GAC TC	58.21	112		
TBP	F	CCC GAA ACG CCG AAT ATA ATC C	59.59	80	100	6
	R	AAT CAG TGC CGT GGT TCG TG	61.23			
TFRC	F	GCGTCGGGATATCGGGTG	60.05	99	100	7
	R	GCAGGATGAAGGGAGGACAC	60.11			

Genes: ribosomal protein L13a (*RPL13A*); actin beta (*ACTB*); eukaryotic translation elongation factor 1 alpha 1 (*EEF1A1)*; hypoxanthine-guanine phosphoribosyltransferase 1 (*HPRT1*); glyceraldehyde-3-phosphate dehydrogenase (*GAPDH*); TATA-box binding protein (*TBP*); transferrin receptor protein (*TFRC*). Primers were obtained from Integrated DNA Technologies, UK.

Supplementary Table 5: Primers used for gene expression analyses

	Gene	F/R	Sequence	Tm (°C)	Amplicon size	Primer Conc. (nM)
Osteogenic	*RUNX2*	F	TCAACGATCTGAGATTTGTGGG	58.40	81	200
		R	GGGGAGGATTTGTGAAGACGG	60.68		
	BGLAP	F	ATGAGAGCCCTCACACTCCT	59.96	117	150
		R	CTTGGACACAAAGGCTGCAC	59.97		
Chondrogenic	*COL2A1*	F	CCAGATGACCTTCCTACGCC	59.89	186	300
		R	TTCAGGGCAGTGTACGTGAAC	60.54		
	SOX9	F	AGCGAACGCACATCAAGAC	58.85	85	150
		R	CTGTAGGCGATCTGTTGGGG	60.18		
Adipogenic	*PPARγ*	F	TACTGTCGGTTTCAGAAATGCC	58.93	141	150
		R	GTCAGCGGACTCTGGATTCAG	60.47		
	C/EBPα	F	GGTGCGTCTAAGATGAGGGG	59.89	141	300
		R	GCATTGGAGCGGTGAGTTTG	60.11		

Genes: runt related transcription factor 2 (*RUNX2*); bone gamma-carboxyglutamate protein (*BGLAP*); collagen type II alpha 1 (*COL2A1*); sex determining region Y (SRY)-box 9 (*SOX9*); peroxisome proliferator-activated receptor gamma (*PPARγ*); CCAAT/enhancer-binding protein alpha (*C/EBPα*). Primers were obtained from Integrated DNA Technologies.

Supplementary Table 6: Antibodies and isotype controls used for immunostaining of hMSC encapsulated within S-HA-PEGDA hydrogels

Target	Antibody	Primary Antibody	Dilution	Product code, Supplier
**Fibronectin**	**Primary**	Mouse monoclonal anti-cellular fibronectin [FN-3E2]	1:350	F6140, Sigma
	**Isotype**	Mouse IgM monoclonal [PFR-03]	1:350	SAB4700727, Sigma
**RUNX2**	**Primary**	Mouse monoclonal anti-RUNX2	1:75	ab76956, Abcam
	**Isotype**	Mouse IgG2a monoclonal [MOPC-173]	1:75	ab18413, Abcam
**PPARγ**	**Primary**	Mouse monoclonal anti-PPARγ	1:100	Sc-7273, Santa Cruz Bitechnology, INC., USA
	**Isotype**	Mouse IgG1 kappa light chain [MOPC-21]	1:100	ab18443, Abcam
	**Secondary**	Goat anti-mouse biotin / streptavidin, AlexaFluor® 488 conjugate	1:300 / 1:500	ab6788, Abcam / S11223, Molecular Probes
**Integrin α_5**	**Primary**	Rabbit polyclonal anti-integrin α_5	1:1000	ab1928, Merck Millipore
**Osteocalcin**	**Primary**	Rabbit polyclonal anti-osteocalcin	1:50	4729, One World Lab Inc., USA
**C/EBPα**	**Primary**	Rabbit IgG polyclonal anti-C/EBPα [14AA)	1:50	sc-61, Santa Cruz Biotechnology, Inc.
	**Isotype**	Rabbit IgG polyclonal	1:1000, 1:50	ab27478, Abcam
	**Secondary**	Alexa Fluor® 647 goat anti-rabbit	1:250	ab150079, Abcam
**Type II collagen**	**Primary**	Rabbit IgG polyclonal anti-collagen II	1:200	ab34712, Abcam
	**Isotype**	Rabbit IgG polyclonal	1:200	ab27478, Abcam
	**Secondary**	Goat polyclonal anti-rabbit biotin / streptavidin, AlexaFluor® 488 conjugate	1:300 / 1:500	ab6720, Abcam / S11223, Molecular Probes
**Tubulin**	**Primary**	Rat IgG2a monoclonal anti-tubulin [YOL1/34]	1:400	ab6161, Abcam
	**Isotype**	Rat IgG2a, κ monoclonal [RTK2758]	1:400	ab18450, Abcam
	**Secondary**	Alexa Fluor® 568 goat anti-rat	1:300	ab175476, Abcam

Supplementary Figures

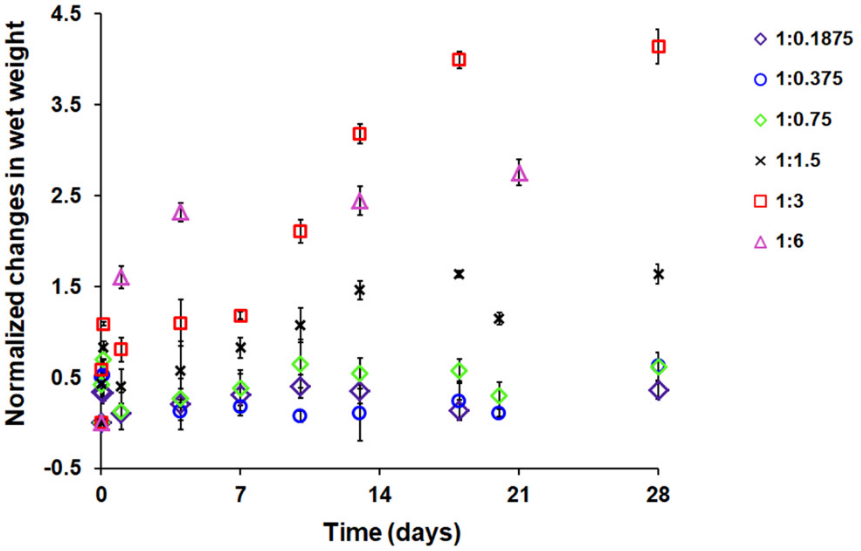

Supplementary Figure 1: Normalized changes in wet weight of hydrogels of different compositions over 28 days incubated in basal culture medium (*n* = 6). Data are shown as mean ± s.d. of the biological replicates. 1:0.75 hydrogels were relatively stable, while hydrogels with lower concentrations of PEGDA contracted (as the cross-linking reaction proceeded) and those with higher concentrations swelled.

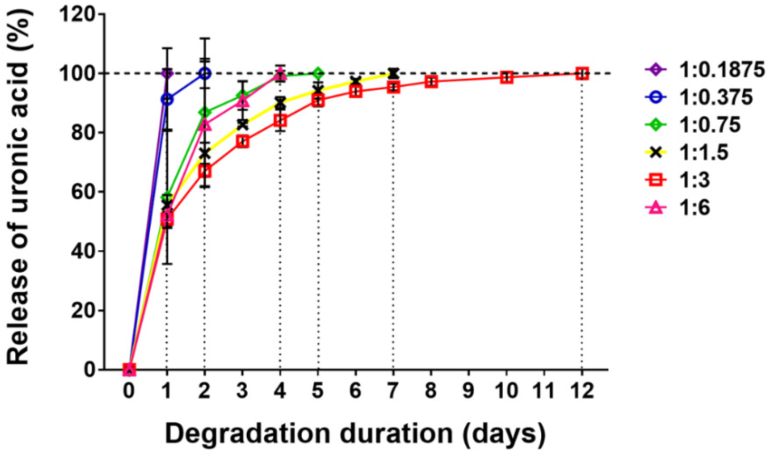

Supplementary Figure 2: Normalized hydrogel degradation over time in the presence of hyaluronidase (100 U mL^{-1}) relative to the PBS control ($n \geq 3$). Data are shown as mean ± s.d. of the biological replicates. Degradation was measured by carbazole assay by quantifying the release of uronic acid. Hydrogels with lower concentrations of PEGDA degraded more quickly than those with higher concentrations of PEGDA.

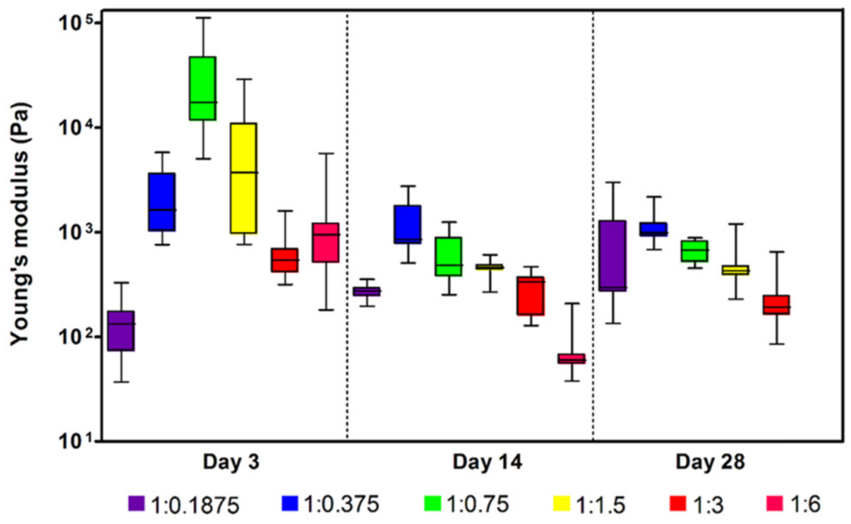

Supplementary Figure 3: Young's modulus (*E*) of acellular S-HA-PEGDA hydrogels as determined by atomic force microscopy (AFM) 3, 14 and 28 days after cross-linking. Values are depicted as medians (central line), 1st/3rd quartiles (bounds of box) and high/low values (whiskers). Measurements were not made immediately after hydrogel formation as the Michael-addition reaction proceeds for some time, further stiffening the hydrogel[12,13]. As others have reported[14], increasing the concentration of cross-linker at a constant S-HA concentration, produces an increase in stiffness up until an optimal concentration after which additional PEGDA results in a reduction in *E*. Over time and in line with swelling and degradation (either by ester hydrolysis or via enzymatic degradation by hyaluronidase, which is present in serum), *E* of all hydrogel compositions decreases (n = 200 per composition, per time point; $p < 0.001$ for all comparisons at every time point by Kruskal-Wallis and Dunn's Multiple Comparison Test). 1:6 hydrogels completely degrade by day 28 so stiffness measurements could not be made.

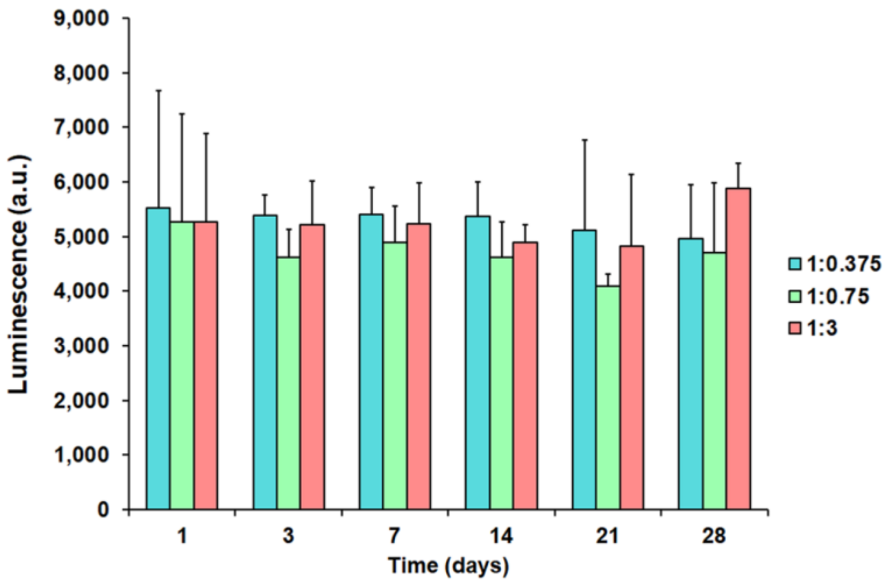

Supplementary Figure 4: hMSC viability, as determined by Real Time-Glo™ MT cell viability assay, in S-HA-PEGDA hydrogels of different compositions seeded at $5x10^5$ cells mL^{-1}. Data are shown as mean + s.d. of the biological replicates (*n* = 3). Although cells remained viable over the 28 day culture period, they did not appear to proliferate. Only 1:0.375, 1:0.75 and 1:3 hydrogels were used in cell experiments as these compositions were both relatively stable in culture and covered a broad range of S-HA to PEGDA weight ratios.

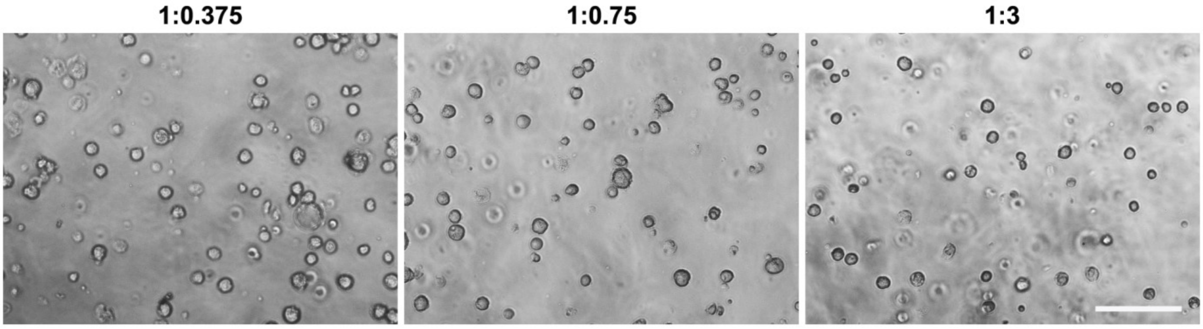

Supplementary Figure 5: Representative micrographs of hMSC ($5x10^5$ cells mL^{-1}) in 1:0.375, 1:0.75 and 1:3 S-HA-PEGDA hydrogels after 24 h in culture. All cells adopted round morphologies, regardless of hydrogel composition. Scale bar = 100 μm.

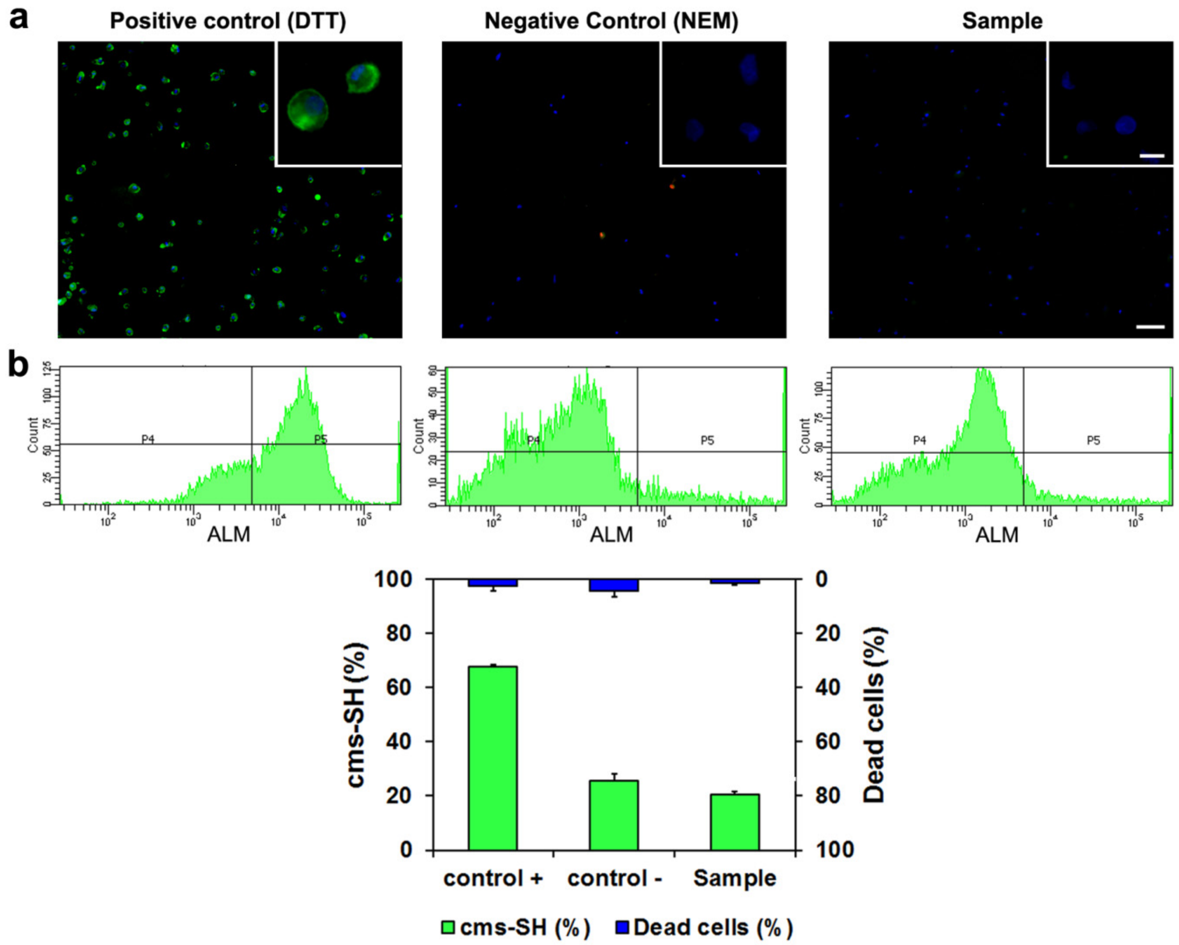

Supplementary Figure 6: a. Representative micrographs of hMSC (2.5x10^5 cells mL^{-1}) treated with dithiothreitol (DTT, positive control), or N-ethylmaleimide (NEM, negative control) or under conditions identical to those used before encapsulation within hydrogels (sample). NEM quenches free –SH groups on the cell surface, blocking labeling by the ALM dye, while DTT cleaves dimerized thiols, making them available for labeling. Cell surface thiols (csm-SH) were detected using Alexa Fluor 488 C5 Maleimide (ALM, green). Cells were also stained with Hoechst 33342 (blue) and ethidium homodimer-1 (red, to detect dead cells). The scale bar represents 100 µm. In insets, the scale bar represents 10 µm. **b**. Quantification by flow cytometry of cell surface thiols (csm-SH, green) and dead cells (DAPI, blue) in hMSC. Data shown are median (%) + s.e.m. of the biological replicates. Quantification analyses show that labeling for sample cells was not different than that of the negative control, confirming that free thiol groups are not available on cell surface before cell encapsulation within hydrogels. This suggests that chemical groups on the cell surface would have few, if any, interactions with components of the hydrogel during the cross-linking reaction. No significant differences were observed in cell viability among conditions tested. Negative control ($p < 0.01$) and sample ($p < 0.001$) showed significantly lower levels of cms-SH than positive control by Kruskal-Wallis and Dunn's Multiple Comparison Test, $n \geq 6$.

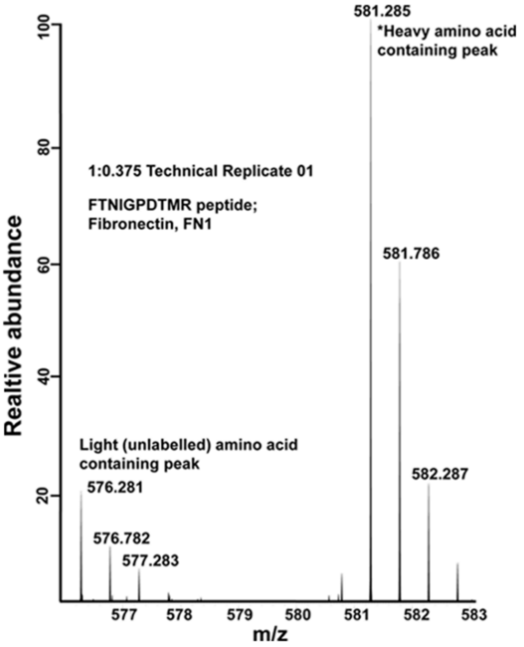

Supplementary Figure 7: Example spectrum for the fibronectin peptide FTNIGPDTMR generated from proteomics analysis. This demonstrate that the heavy SILAC label was incorporated into proteins synthesized by hMSC post encapsulation. The peptide contains one arginine and therefore the mass difference between the heavy and light peak is 10 Da.

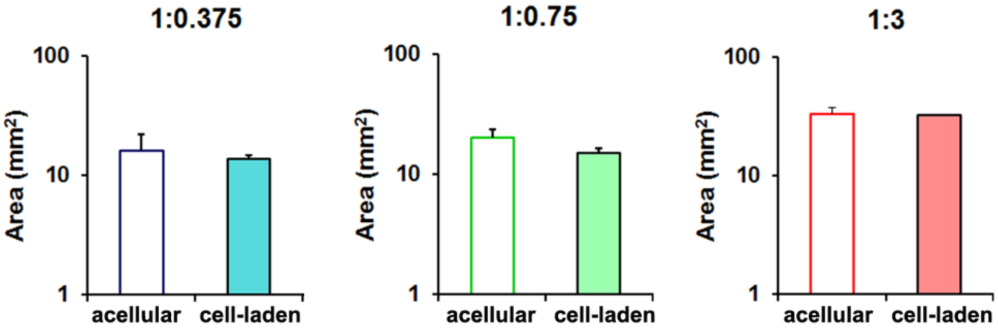

Supplementary Figure 8: Bulk changes in hydrogel size determined by measuring hydrogel cross-sectional area after 72 h in culture. S-HA-PEGDA hydrogels were either acellular or cell-laden (5x10^5 cells mL^{-1}). Data are shown as mean + s.d. of the biological replicates ($n \geq 3$).

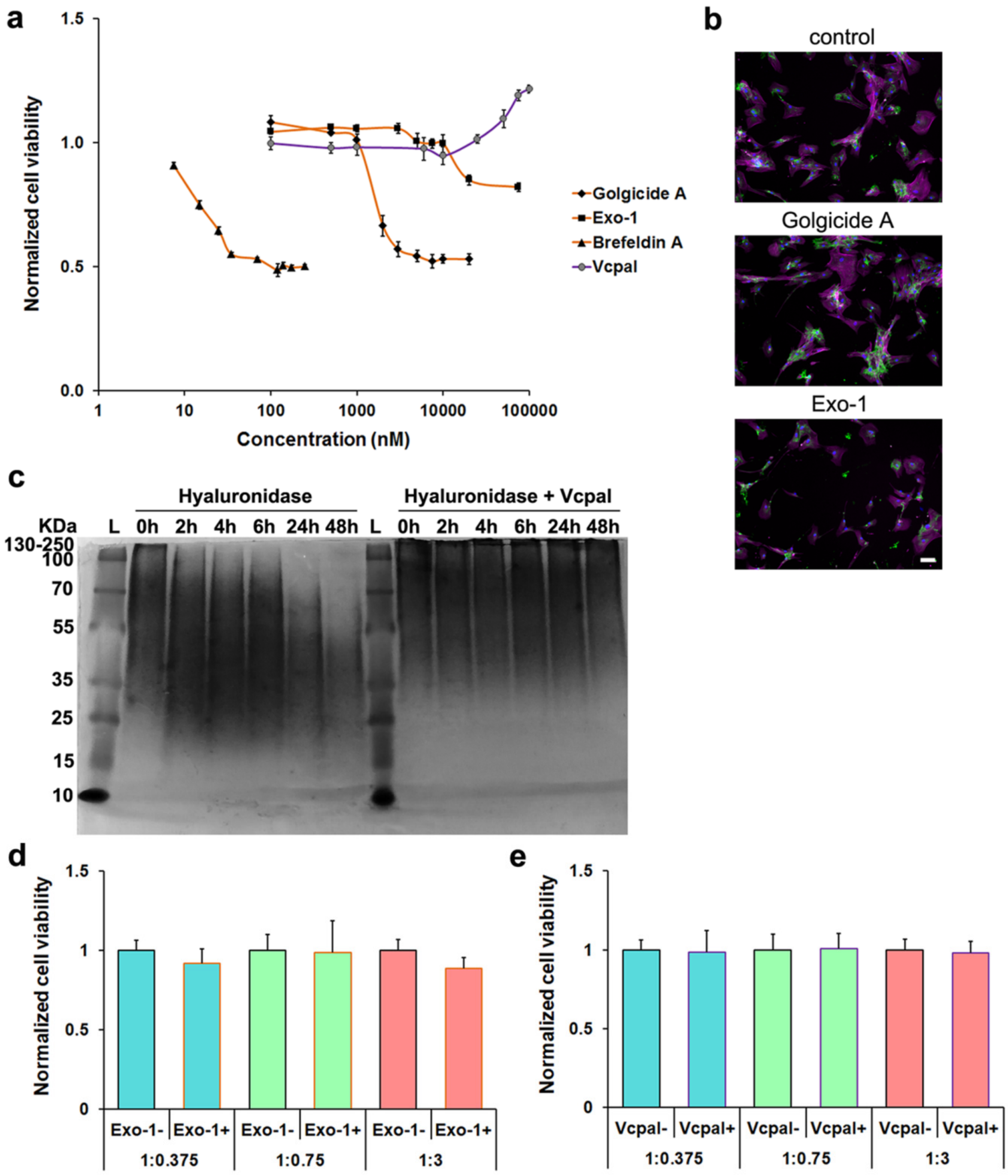

Supplementary Figure 9: **a**. Cell viability determined by alamarBlue® activity of hMSC (45,000 cells per cm^2 on TCP, $n \geq 10$) treated for 72 h with Vcpal (0.1-100 µM), Golgicide A (0.1-30 µM), Brefeldin A (7.5-500 nM) and Exo-1 (0.1-75 µM). **b**. Representative micrographs of fibronectin (green) secreted by hMSC that were either untreated (control) or treated with Golgicide A (1 µM) or Exo-1 (75 µM). Exo-1 inhibited the secretion of fibronectin to a qualitatively greater extent than did Golgicide A, at the non-toxic concentrations tested. Actin is stained with phalloidin-TRITC (magenta) and nuclei are counterstained with DAPI (blue). Scale bar = 100 µm. **c**. Stains-All gel showing S-HA fragments produced after incubation for up to 48 h with hyaluronidase (100 U mL^{-1}) or hyaluronidase combined with Vcpal (100 µM). Hyaluronidase degrades S-HA to small fragments, but in the presence of Vcpal, hyaluronidase activity is inhibited, and small fragments are not evident. L designates

the ladder. Cell viability, as determined by Real Time-Glo^{TM} MT cell viability assay (*n* = 3), of hMSC encapsulated within S-HA-PEGDA hydrogels of different compositions and treated for 72 h with either **d**. Exo-1 (75 μM) or **e**. Vcpal (100 μM). In **a**., **d**., and **e**. data are shown as mean + s.d. of the biological replicates.

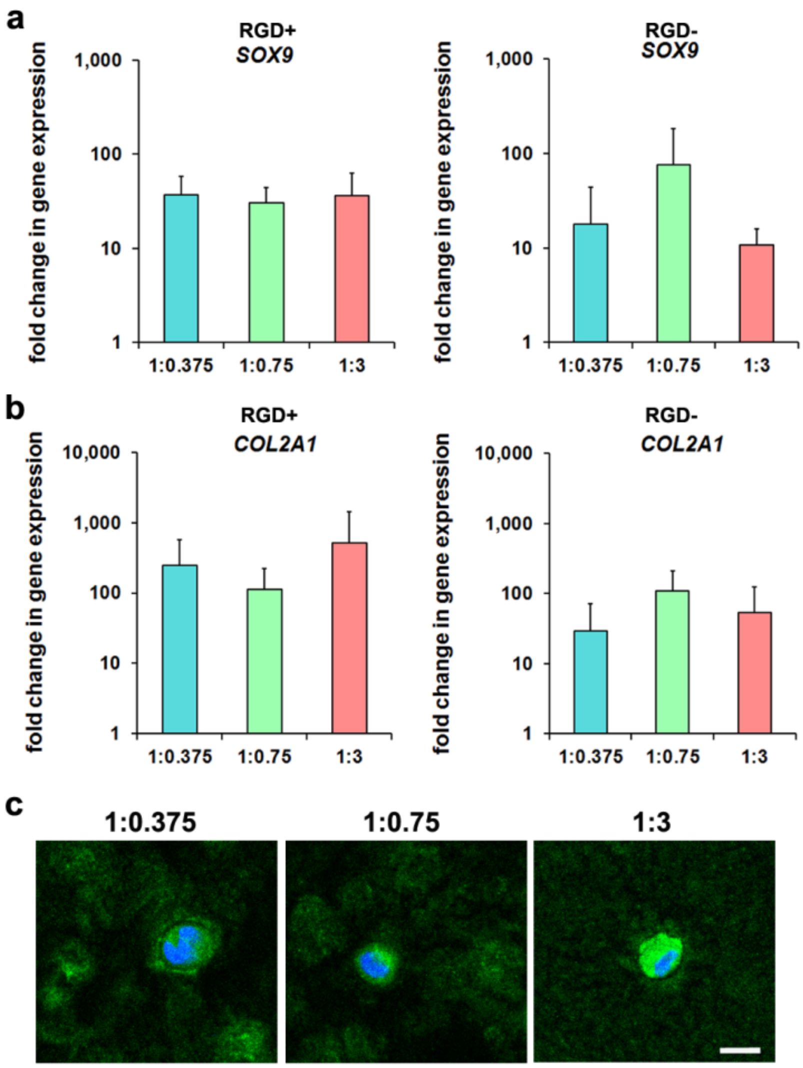

Supplementary Figure 10: **a**. Gene expression analyses for *SOX9*, a marker of chondrogenesis, and **b**. *COL2A1*, the most abundant protein in cartilage, in hMSC 72 h after encapsulation in 1:0.375, 1:0.75 and 1:3 S-HA-PEGDA hydrogels cultured with RGD sequence-containing peptides (RGD+) or with a scrambled control peptide (RGD-). Expression levels are shown as fold change normalized to expression in undifferentiated hMSC (set to 1). Data are shown as mean + s.d. of the biological replicates. Expression of both genes was significantly upregulated in all groups compared to undifferentiated hMSC controls suggesting a chondrogenic effect of the HA-based matrix ($n \geq 6$). The addition of RGD sequence-containing peptides in comparison to the RGD- control significantly affected the expression of *SOX9* when hMSC were encapsulated in 1:0.375 ($p < 0.05$) and 1:3 ($p < 0.001$) hydrogels, and *COL2A1* when encapsulated in 1:0.375 ($p < 0.01$) hydrogels; however, all other comparisons were not significant by Kruskal-Wallis and Dunn's Multiple Comparison Test. Nevertheless, both *SOX9* and *COL2A1* were still significantly upregulated in all groups compared to undifferentiated controls. Moreover, we could not detect any hydrogel composition-dependent differences in expression in the presence of RGD sequence-containing peptides. Taken together, these observations suggest that the HA matrix itself, rather than integrin-mediated interactions with a secreted pericellular matrix drives hMSC differentiation towards the chondrogenic lineage. **c**. Immunostaining for type II collagen (green) and DAPI (blue) of hMSC encapsulated within 1:0.375, 1:0.75 and 1:3 S-HA-PEGDA hydrogels and cultured for 28 days. Positive staining is evident around hMSC

encapsulated within all hydrogel compositions. At least 20 cells per conditions were examined. Scale bar = 10 μm.

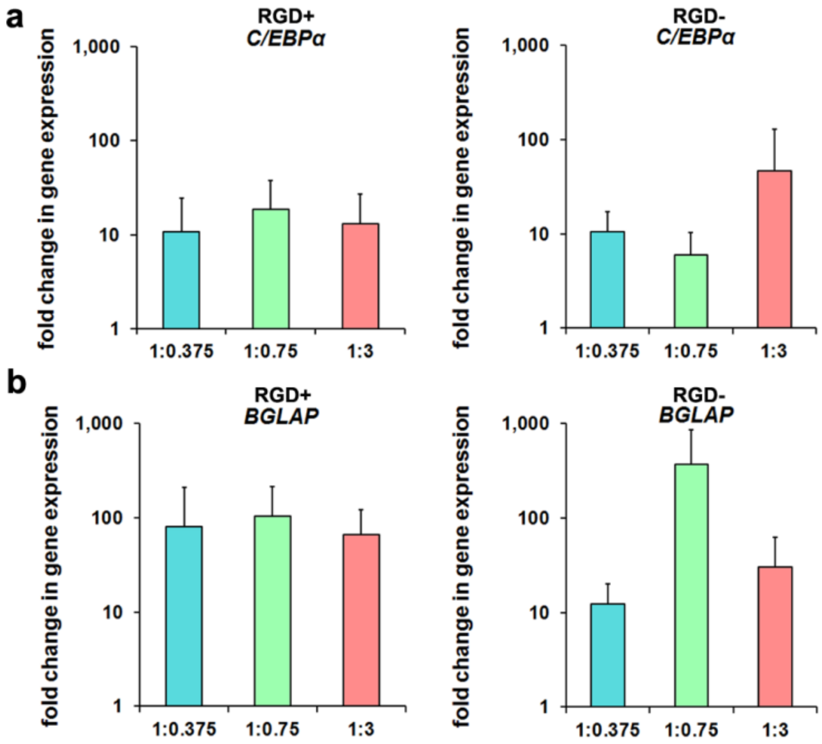

Supplementary Figure 11: **a**. Gene expression analysis for *C/EPBα*, which encodes a transcription factor that acts with *PPARγ* in adipogenesis, in hMSC 72 h after encapsulation in 1:0.375, 1:0.75 and 1:3 S-HA-PEGDA hydrogels cultured with RGD sequence-containing peptides (RGD+) or with a scrambled control peptide (RGD-). Expression levels are shown as fold change normalized to expression in undifferentiated hMSC (set to 1). Trends (although not significant, by Kruskal-Wallis and Dunn's Multiple Comparison Test, $n \geq 6$) for increased expression of *C/EPBα* in hMSC in 1:0.375 and 1:3 hydrogels were eliminated in cultures treated with RGD sequence-containing peptides. **b**. Gene expression analyses for *BGLAP*, which encodes the bone-specific protein osteocalcin, in hMSC 72 h after encapsulation in 1:0.375, 1:0.75 and 1:3 S-HA-PEGDA hydrogels cultured with RGD sequence-containing peptides (RGD+) or with a scrambled control peptide (RGD-). hMSC in 1:0.75 showed a trend for increased expression compared to those in 1:0.375 and 1:3 hydrogels. This effect was eliminated in cultures treated with RGD sequence-containing peptides (Kruskal-Wallis and Dunn's Multiple Comparison Test, $n \geq 6$). Data are shown as mean + s.d. of the biological replicates.

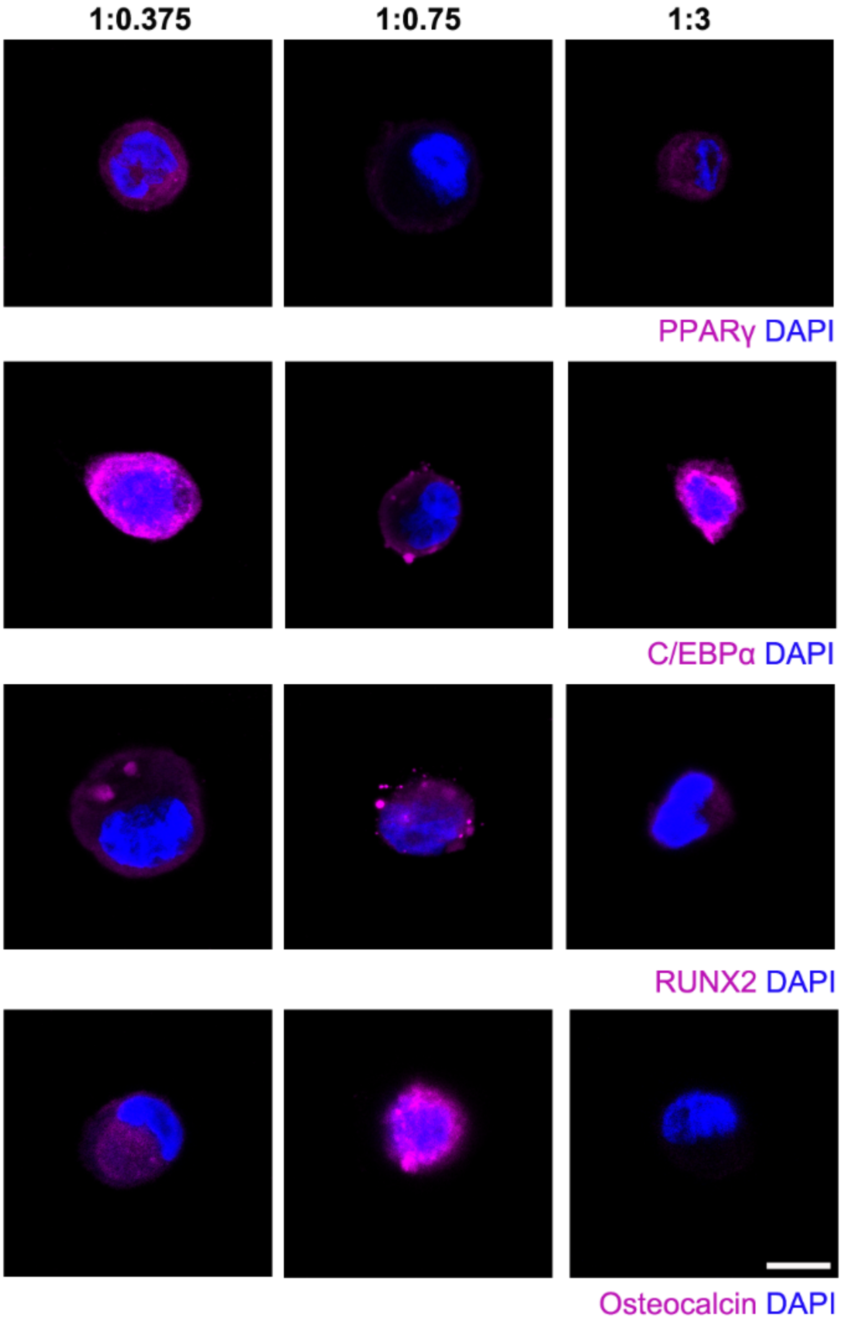

Supplementary Figure 12: Representative micrographs of immunostaining for PPARγ, C/EPBα, RUNX2, and osteocalcin in hMSC within S-HA-PEGDA hydrogels after culture in basal medium for 72 h. Extensive positive nuclear staining for adipogenic markers PPARγ and C/EBPα was evident in hMSC encapsulated within 1:0.375 and 1:3 hydrogels, while positive staining for RUNX2 (nuclear) and osteocalcin was evident in hMSC encapsulated in 1:0.75 hydrogels. All images show staining for DAPI (blue). Scale bar = 10 μm.

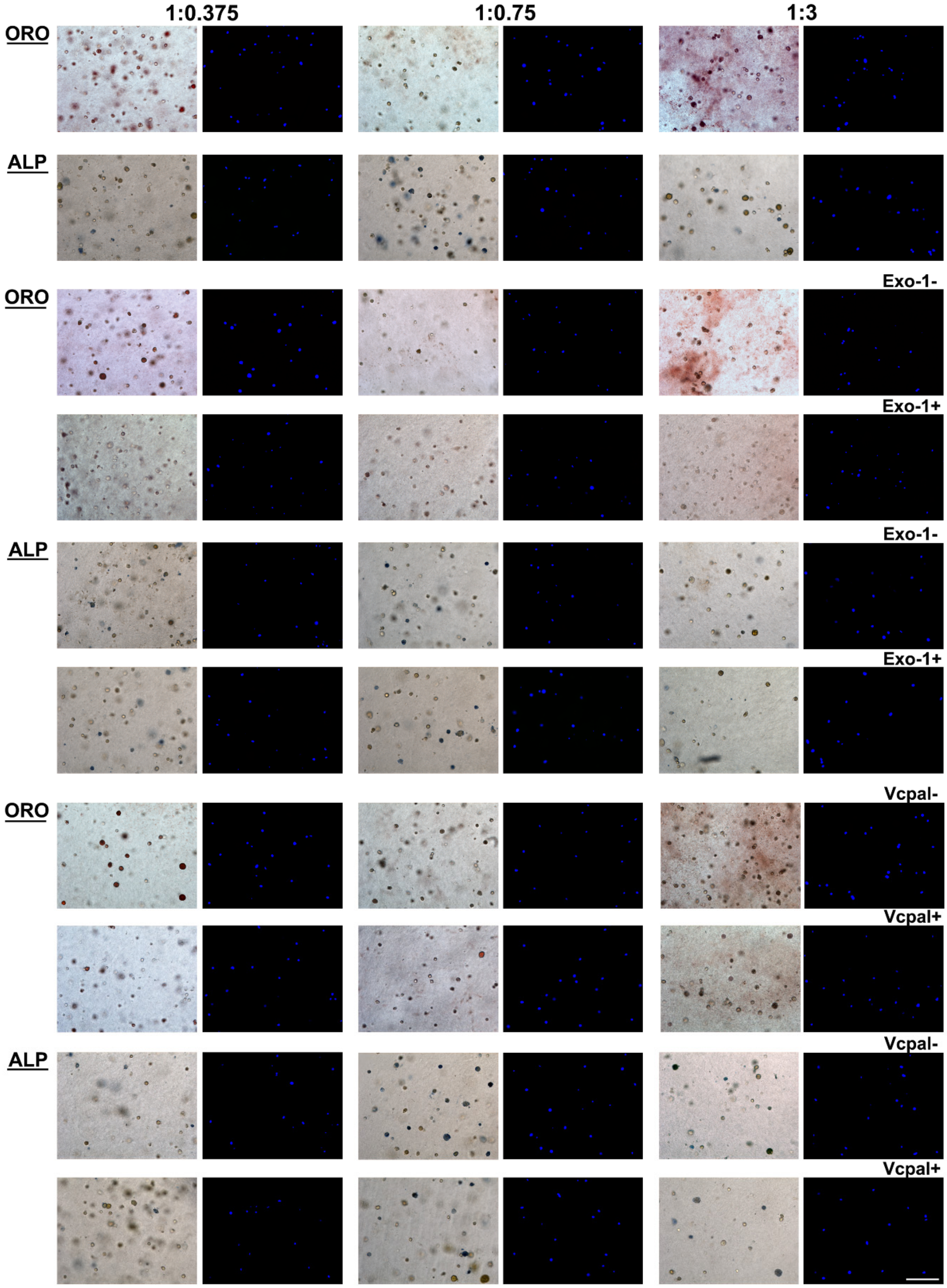

Supplementary Fig. 13: Representative micrographs showing histochemical staining for Oil Red O (ORO) and alkaline phosphatase (ALP) and accompanying DAPI (blue) images of hMSC cultured in 1:0.375, 1:0.75 and 1:3 S-HA-PEGDA hydrogels for 14 days in a bi-potential osteogenic/adipogenic culture medium. Images either show ORO and ALP staining

under standard conditions or treated with 75 µM Exo-1 or 100 µM Vcpal (Exo-1+/Vcpal+) or their respective vehicle controls (Exo-1-/Vcpal-). Scale bar = 100 µm.

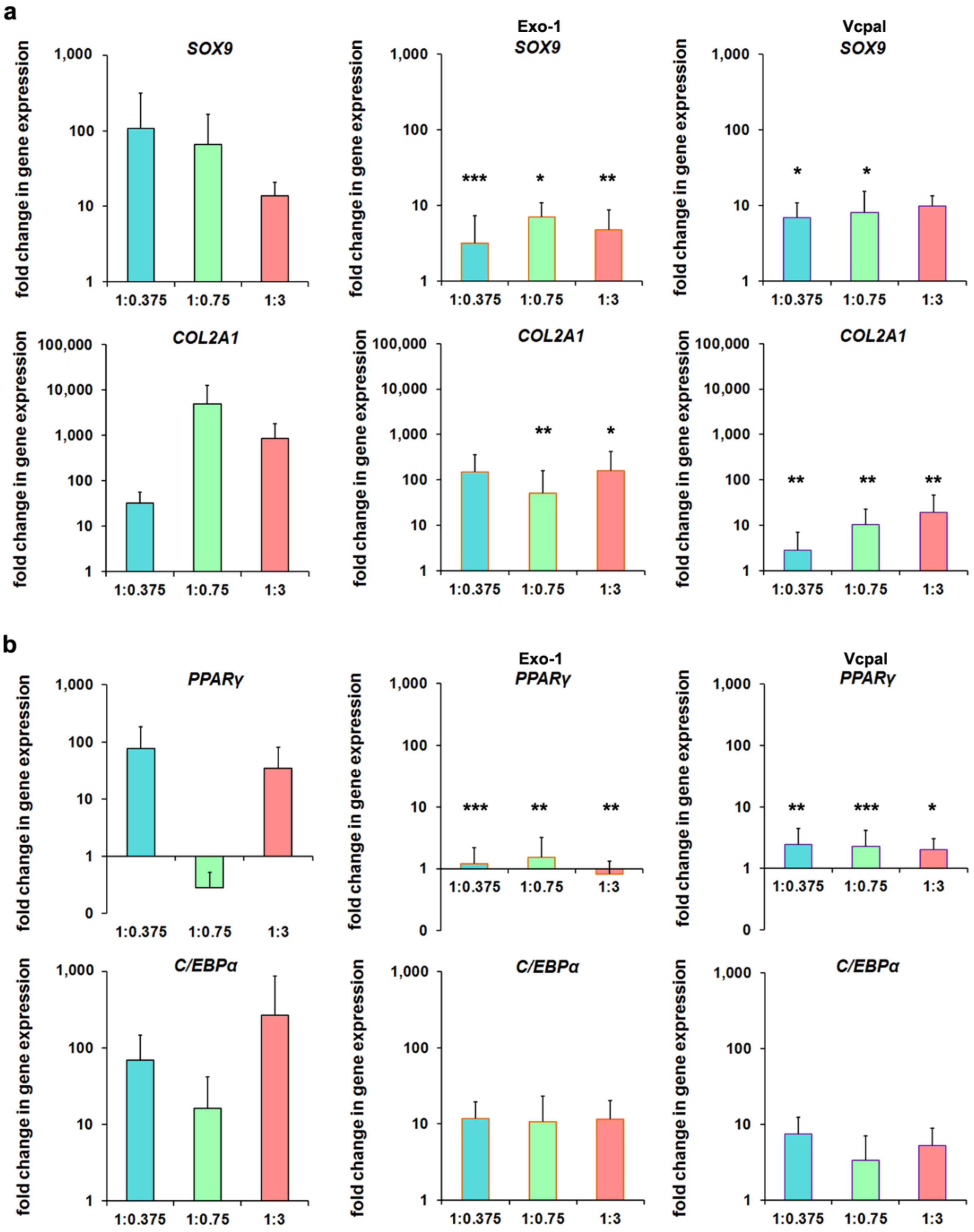

a
SOX9
Exo-1
SOX9
Vcpal
SOX9
COL2A1
COL2A1
COL2A1
fold change in gene expression
1:0.375
1:0.75
1:3
b
PPARγ
Exo-1
PPARγ
Vcpal
PPARγ
C/EBPα
C/EBPα
C/EBPα

c

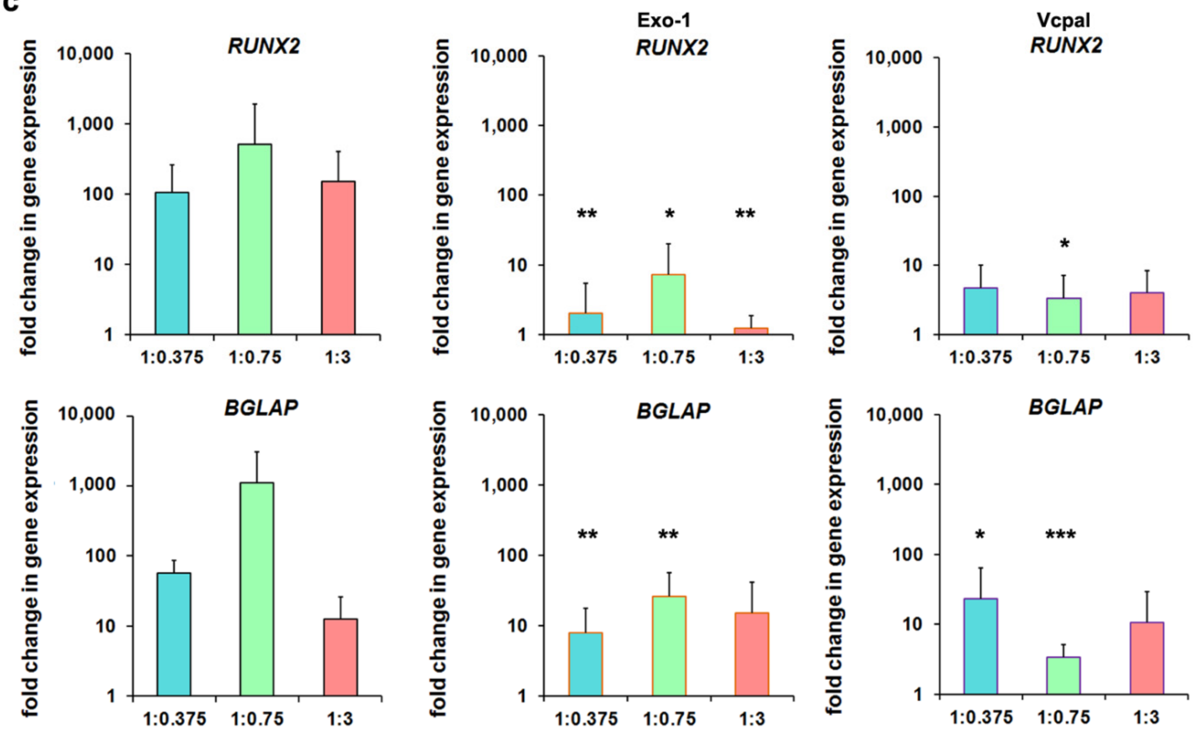

Supplementary Figure 14: **a**. Gene expression analyses for markers of chondrogenesis (*SOX9* and *COL2A1*), **b**. adipogenesis (*PPARγ* and *C/EPBα*), and **c**. osteogenesis (*RUNX2* and *BGLAP*) in hMSC 72 h after encapsulation in 1:0.375, 1:0.75 and 1:3 S-HA-PEGDA hydrogels (5×10^5 cells mL^{-1}) cultured with basal culture medium (vehicle control, left) or treated with 75 µM Exo-1 (middle, outlined orange) or treated with 100 µM Vcpal (right, outlined purple). Expression levels are shown as fold change normalized to expression in undifferentiated hMSC (set to 1). Treated conditions were compared with the vehicle using a Mann-Whitney test (two-tailed, $n \geq 6$), *$p < 0.05$, **$p < 0.01$, ***$p < 0.001$. *SOX9* and *COL2A1* expression were reduced but still upregulated using both inhibitors in cell-laden hydrogels of different compositions suggesting a chondrogenic effect of the HA-based matrix. The strong upregulation of *PPARγ* in 1:0.375 and 1:3 hydrogels was significantly reduced upon treatment with Exo-1. Trends (although not significant) for decreased expression of *C/EPBα* in 1:0.375 and 1:3 hydrogels were also observed. Similarly, the strong upregulation of osteogenesis markers *BGLAP* and *RUNX2* in 1:0.75 hydrogels was abrogated by treatment with Vcpal. Significant downregulation of *PPARγ* expression in 1:0.375 and 1:3 hydrogels, for example, after treatment with Vcpal suggests that degradation is necessary to drive pericellular matrix formation and lineage specification. Data are shown as mean + s.d. of the biological replicates.

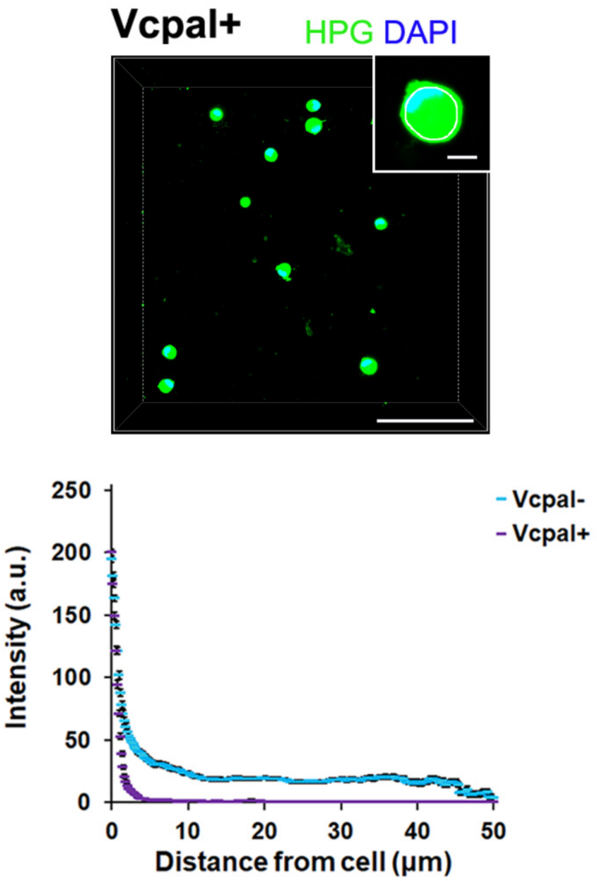

Supplementary Fig. 15: Representative micrographs showing fluorescence labeling of the methionine analogue L-homopropargylglycine (HPG, green) in hMSC-laden hydrogels (1:0.375) treated with 100 μM Vcpal after 72 h. The cell membrane, as determined by DIC imaging, is outlined in white. Scale bar = 100 μm, inset = 10 μm. Plots show mean intensity of the fluorescence signal (± s.e.m) as a function of distance from the cell membrane under control conditions (Vcpal-, blue dashes) or when treated with Vcpal (Vcpal+, purple dashes, n = 30 cells per condition, 40 plots per cell). Inhibiting hyaluronidase activity with Vcpal impacted the distance at which protein was detected around cells. Under control conditions (Vcpal-), proteins were detected up to 50 μm from the cell membrane; however, in the presence of Vcpal, proteins were only detected in close proximity to the cell membrane (~5 μm).

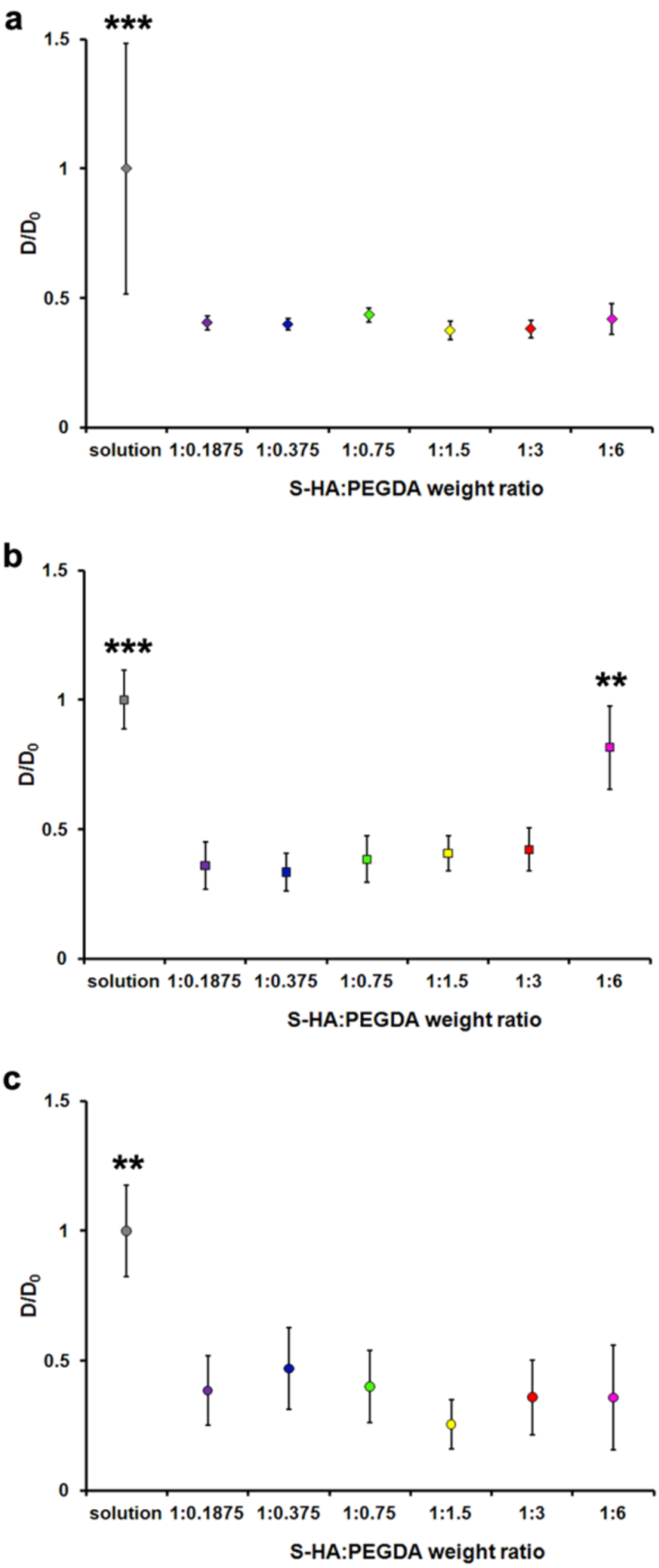

Supplementary Figure 16: Fluorescence recovery after photobleaching (FRAP) measurements of hydrogel diffusivity (D), relative to diffusion within the solution alone (D_o). For dextran sizes of **a.** 70 kDa, **b.** 500 kDa and **c.** 2000 kDa, there were significant differences in D/D_o between all hydrogel compositions and the solution alone ($n = 4$, $p < 0.01$

by Kruskal-Wallis and Dunn's Multiple Comparison Test). Nevertheless, we could not detect significant differences in D/D_o amongst any of the hydrogel compositions, with the exception of 1:6 hydrogels, which had a significantly higher D/D_o compared to all other hydrogel compositions when tested with the 500 kDa Dextran ($n = 4$, $p < 0.01$ by Kruskal-Wallis and Dunn's Multiple Comparison Test). Data are shown as mean + s.d. of the biological replicates.

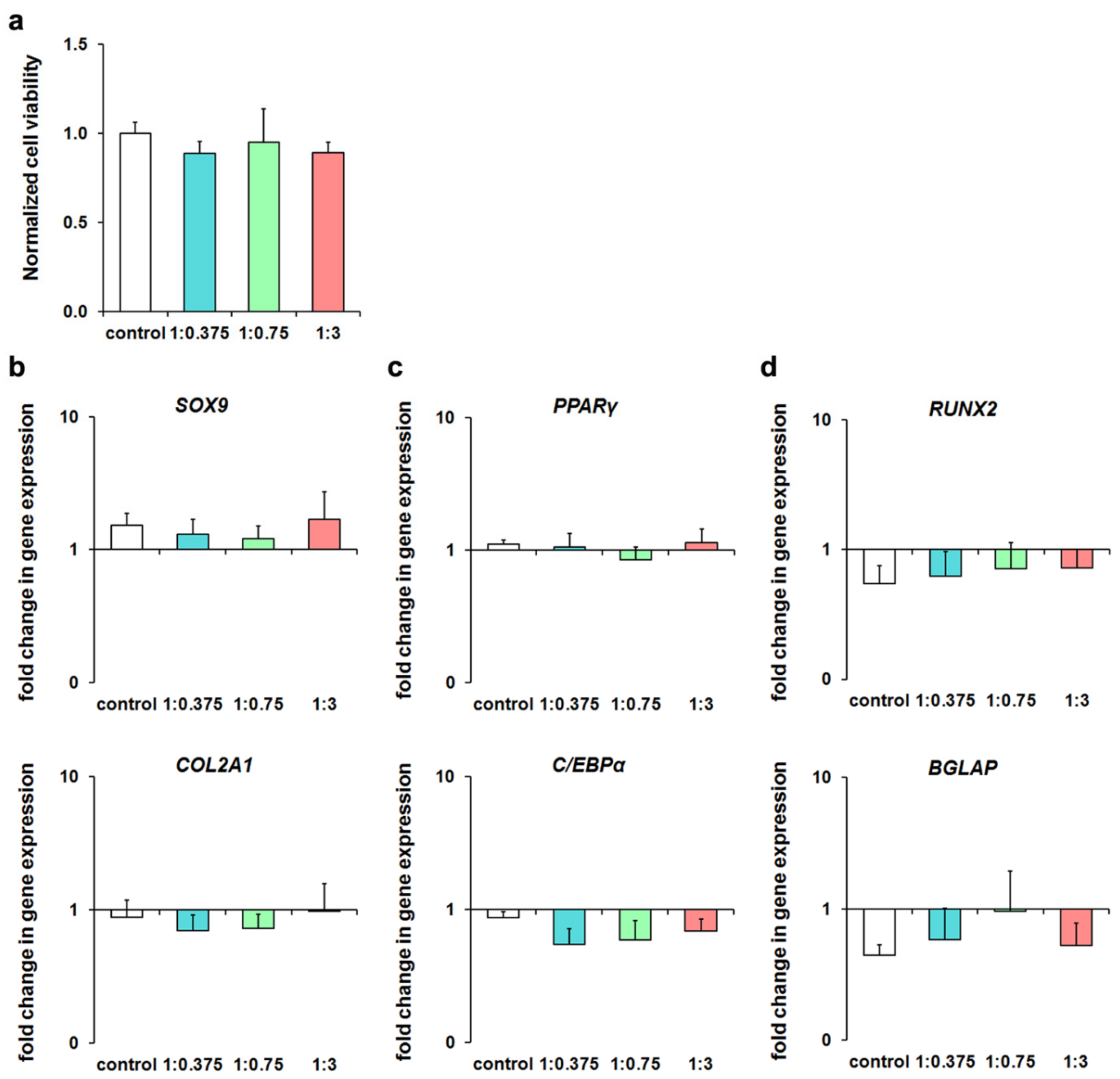

Supplementary Figure 17: **a**. Cell metabolic activity, as determined by alamarBlue® activity, was not significantly different after 3 days (13,500 cells per cm^2) in contact with fragments of S-HA-PEGDA hydrogels of different compositions or with a control solution of 2000 U mL^{-1} hyaluronidase 1 and 2 (n = 5, by Kruskal-Wallis and Dunn's Multiple Comparison Test). Gene expression analyses for **b**. chondrogenesis markers (*SOX9* and *COL2A1*), **c**. adipogenesis markers (*PPARγ* and *C/EPBα*), and **d.** osteogenesis markers (*RUNX2* and *BGLAP*) showed no significant differences in expression (13,500 cells per cm^2) when cultured in contact with fragments of S-HA-PEGDA hydrogels of different compositions or compared to control solutions containing 2000 U mL^{-1} inactivated hyaluronidase after 3 days of exposure (n = 5, by Kruskal-Wallis and Dunn's Multiple Comparison Test). Data are shown as mean + s.d. of the biological replicates.

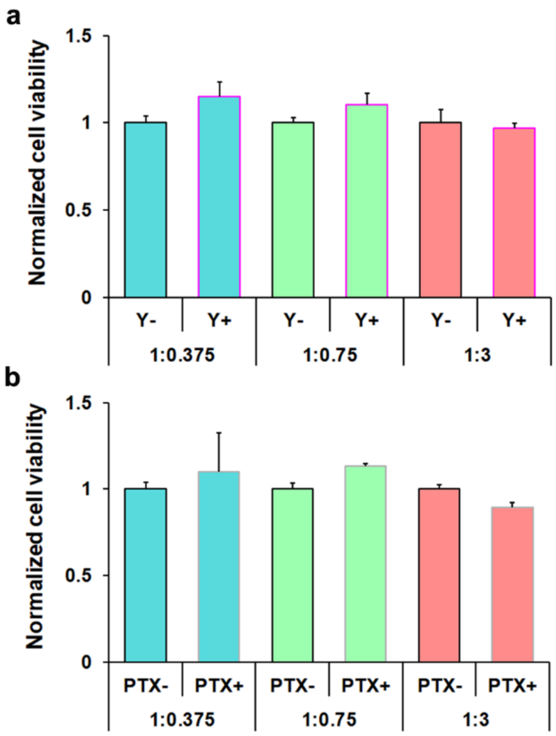

Supplementary Figure 18: Cell viability, as determined by Real Time-Glo^{TM} MT cell viability assay, of hMSC ($5x10^5$ cells mL^{-1}) encapsulated within S-HA-PEGDA hydrogels of different compositions and treated for 72 h with either **a**. Y-27632 (+Y, 10 μM) or **b**. paclitaxel (+PTX, 50 nM). Data are shown as mean + s.d. of the biological replicates (n = 3).

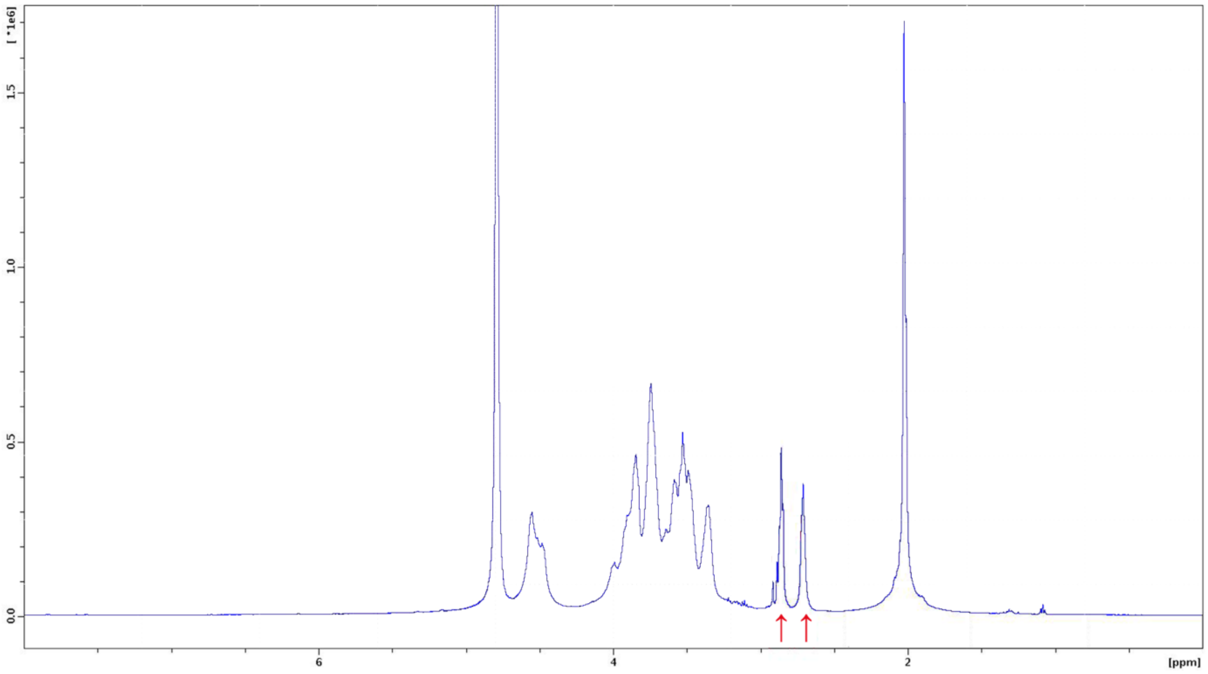

Supplementary Figure 19: Representative ^{1}H NMR spectrum of S-HA. Resonances at 2.8 and 2.7 ppm (arrows in red) indicate the successful thiolation of HA.

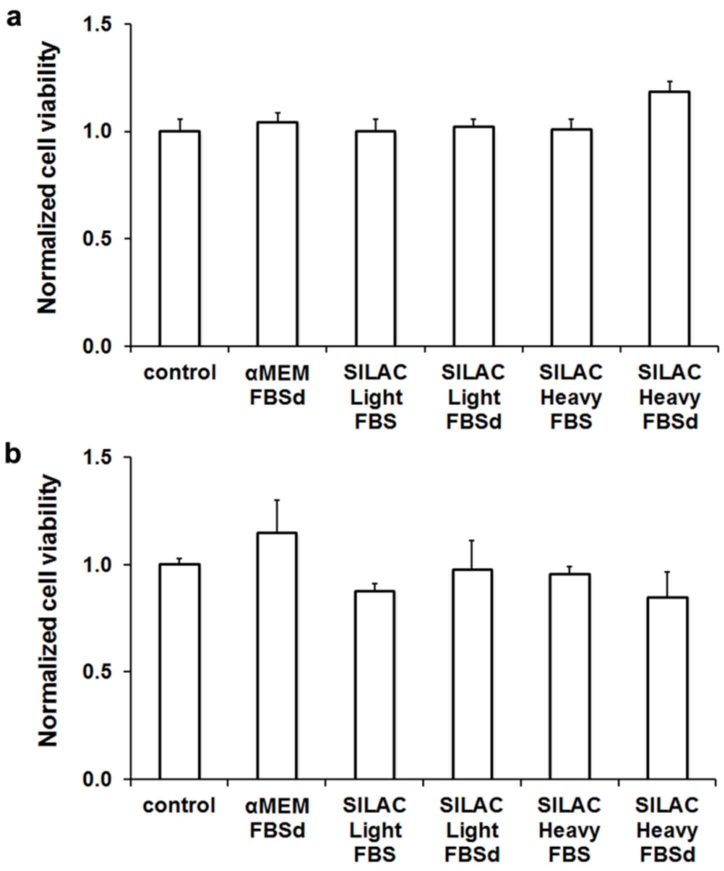

Supplementary Figure 20: **a**. Cell viability of hMSC cultured on tissue culture plastic as determined by alamarBlue® activity (45,000 cells per cm^2 in TCP, $n \geq 10$) or **b**. within hydrogels ($5x10^5$ cells mL^{-1}, 1:0.75 hydrogels, $n = 3$) as determined by Real Time-Glo™ MT cell viability assay. Assays were carried out after treating cultures for 3 days with either basal culture medium (control), αMEM with 10% dialyzed FBS (αMEM FBSd), SILAC with "light" amino acids with 10% FBS (SILAC Light FBS), SILAC with "light" amino acids with 10% dialyzed FBS (SILAC Light FBSd), SILAC with "heavy" amino acids with 10% of FBS (SILAC Heavy FBS), or SILAC with "heavy" amino acids with 10% dialyzed FBS (SILAC Heavy FBSd). All media were supplemented with 1% antibiotic-antimycotic solution. On tissue culture plastic (**a**.), only SILAC Heavy FBSd showed significantly higher viability than the control ($p < 0.001$ by Kruskal-Wallis and Dunn's Multiple Comparison Test, $n \geq 10$). Data are shown as mean + s.d. of the biological replicates.

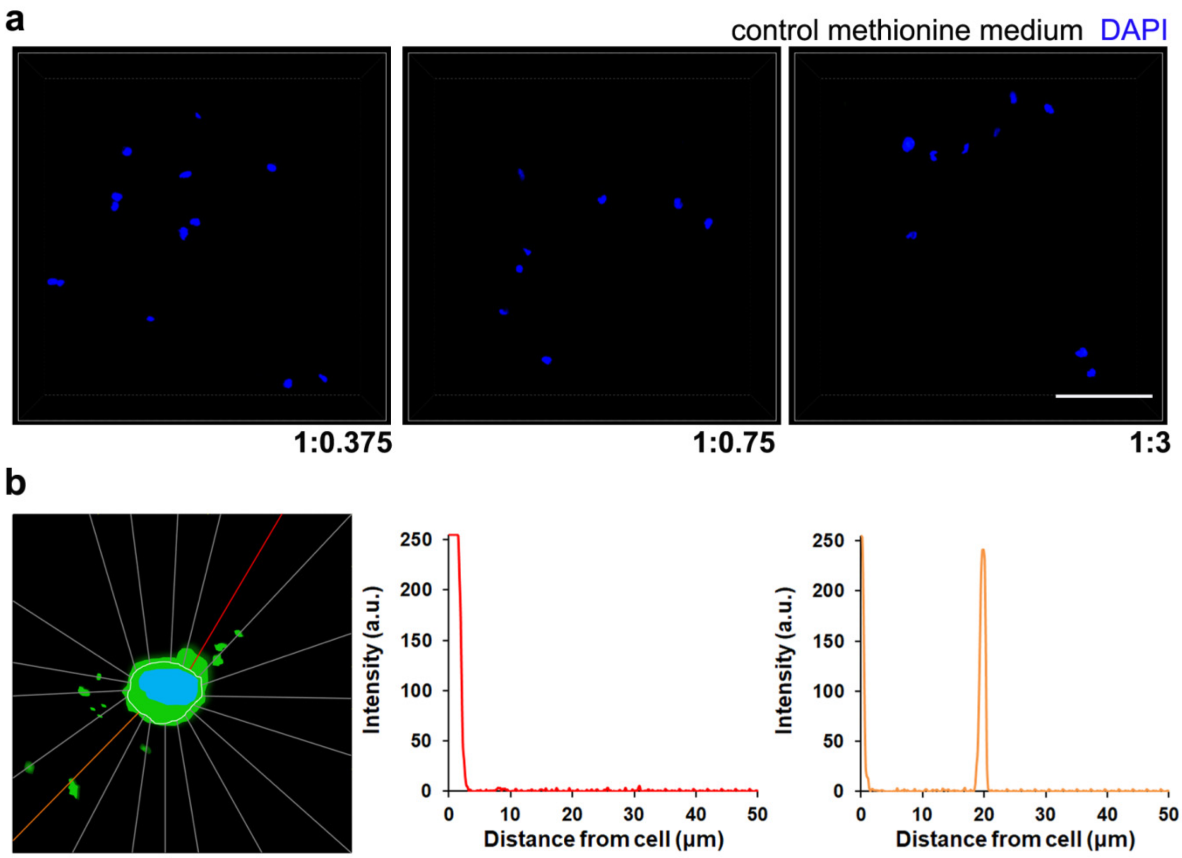

Supplementary Figure 21: **a**. Representative micrographs from control experiments using the fluorescent non-canonical amino acid tagging technique to analyze secreted proteins. Images show cultures of hMSC in 1:0.375, 1:0.75 and 1:3 S-HA-PEGDA hydrogels after 72 h in control methionine media (no L-homopropargylglycine). Fluorescence tagging with the Alexa Fluor 488 azide produced no positive staining. Scale bar = 100 μm. **b**. Schematic highlighting how fluorescence labeling of the methionine analogue L-homopropargylglycine (green) in hMSC-laden hydrogels was quantified. The cell membrane, as determined by DIC imaging, is outlined in white. Profile plots were randomly drawn around the cell in 2 central Z-slices (grey lines) per cell (generating 40 profile plots per cell) and pixel intensity was determined using the multi plot plugin in Image J. 30 cells were analyzed per condition. Representative plots (corresponding to red and orange lines) show example measurements of fluorescence intensity signal (arbitrary units) as a function of distance from the cell membrane.

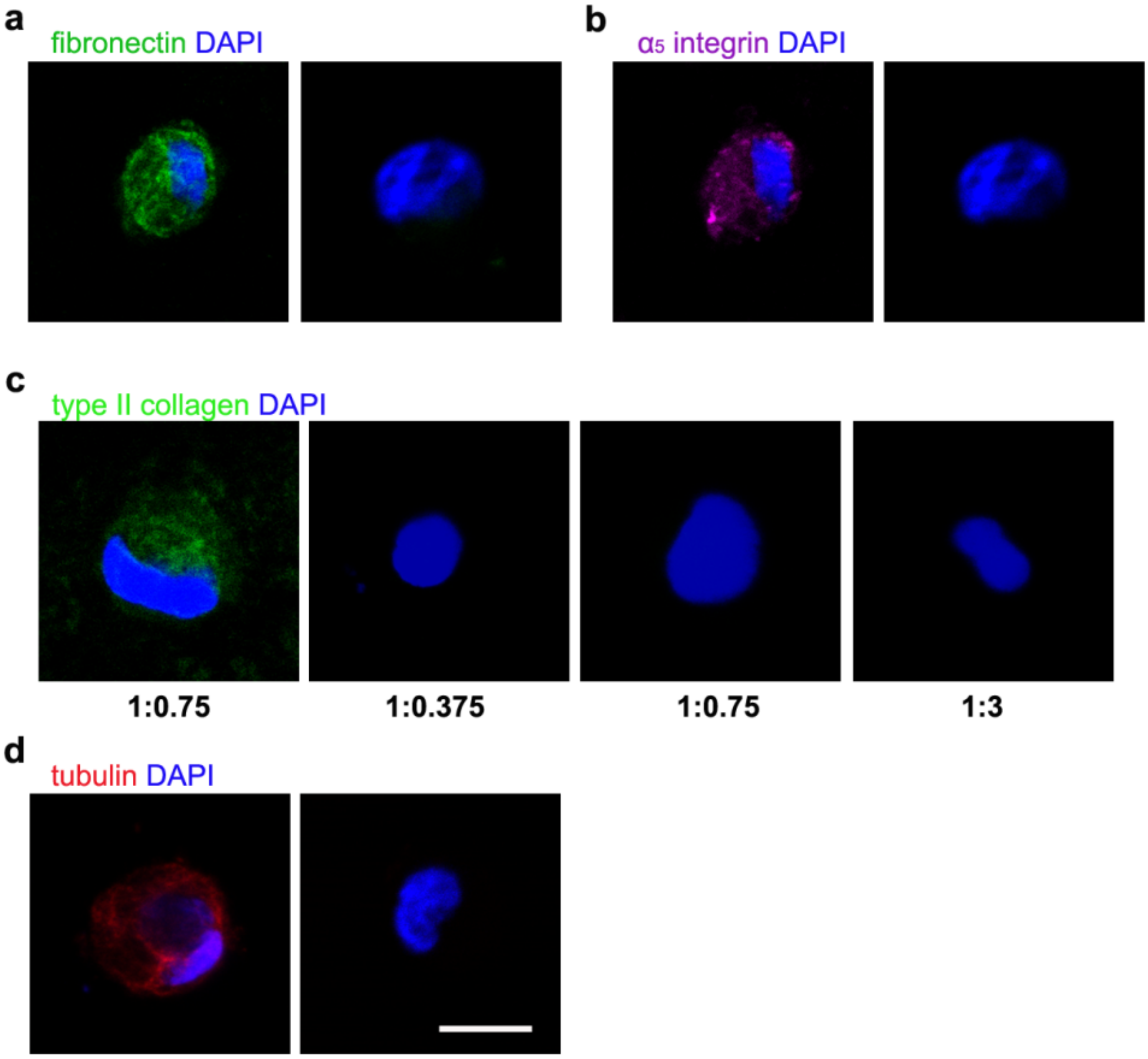

Supplementary Figure 22: Representative micrographs for **a.** fibronectin, **b.** α5 integrin, **c.** type II collagen, and **d.** tubulin of hMSC-laden hydrogels immunostained with primary antibodies (left) and their respective isotype controls (right) followed by appropriate secondary antibody staining. All images show staining for DAPI (blue). Scale bar = 10 μm.

Supplementary references

1. Chung, C., Beecham, M., Mauck, R. L. & Burdick, J. A., The influence of degradation characteristics of hyaluronic acid hydrogels on in vitro neocartilage formation by mesenchymal stem cells. *Biomaterials* **30,** 4287-4296 (2009).
2. Frazier, S. B., Roodhouse, K. A., Hourcade, D. E. & Zhang, L., The Quantification of Glycosaminoglycans: A Comparison of HPLC, Carbazole, and Alcian Blue Methods. *Open glycoscience* **1,** 31-39 (2008).
3. Kang, M., Day, C. A., Kenworthy, A. K. & DiBenedetto, E., Simplified equation to extract diffusion coefficients from confocal FRAP data. *Traffic* **13,** 1589-1600 (2012).
4. Sahaf, B., Heydari, K., Herzenberg, L. A. & Herzenberg, L. A., Lymphocyte surface thiol levels. *Proc. Natl. Acad. Sci. USA* **100,** 4001-4005 (2003).
5. Liu, M. J., Li, X. L. & Hassel, B. A., Proteasomes modulate conjugation to the ubiquitin-like protein, ISG15. *J. Biol. Chem.* **278,** 1594-1602 (2003).
6. Manders, E. M. M., Verbeek, F. J. & Aten, J. A., Measurement of colocalization of objects in dual-color confocal images. *J. Microsc.* **169,** 375-382 (1993).
7. Chung, C. & Burdick, J. A., Influence of three-dimensional hyaluronic acid microenvironments on mesenchymal stem cell chondrogenesis. *Tissue eng. Part A* **15,** 243-254 (2009).
8. Steward, A. J., Wagner, D. R. & Kelly, D. J., The pericellular environment regulates cytoskeletal development and the differentiation of mesenchymal stem cells and determines their response to hydrostatic pressure. *Eur. Cells Mater.* **25,** 167-178 (2013).
9. Stern, R., Asari, A. A. & Sugahara, K. N., Hyaluronan fragments: an information-rich system. *Eur. J. Cell Biol.* **85,** 699-715 (2006).
10. Stern, R. & Jedrzejas, M. J., Hyaluronidases: Their genomics, structures, and mechanisms of action. *Chem. Rev.* **106,** 818-839 (2006).
11. Gerecht, S. *et al.*, Hyaluronic acid hydrogel for controlled self-renewal and differentiation of human embryonic stem cells. *Proc. Natl. Acad. Sci. USA* **104,** 11298-11303 (2007).
12. Young, J. L. & Engler, A. J., Hydrogels with time-dependent material properties enhance cardiomyocyte differentiation in vitro. *Biomaterials* **32,** 1002-1009 (2011).
13. Rehfeldt, F. *et al.*, Hyaluronic acid matrices show matrix stiffness in 2D and 3D dictates cytoskeletal order and myosin-II phosphorylation within stem cells. *Integr Biol-Uk* **4,** 422-430 (2012).
14. Prestwich, G. D., Simplifying the extracellular matrix for 3D cell culture and tissue engineering: A pragmatic approach. *J. Cell. Biochem.* **101,** 1370-1383 (2007).
