# Supplementary material for: Bi-directional cell-pericellular matrix interactions direct stem cell fate
Source: Nat Commun. 2018 Oct 3;9:4049. doi: 10.1038/s41467-018-06183-4 (PMC6170409; doi:10.1038/s41467-018-06183-4)
Supplement: Supplementary file 2 — Description of Additional Supplementary Files [file 41467_2018_6183_MOESM2_ESM.pdf]

Description of Additional Supplementary Files

File Name: Supplementary Data 1
Description: List of pericellular matrix components detected by proteomic analysis that were synthesized and secreted by hMSC, and maintained within S-HA-PEGDA 1:0.375, 1:0.75 and 1:3 hydrogels. Heatmap shows the percentage of heavy label after 1, 2 and 3 days in SILAC media in technical replicates (hMSC treated either from encapsulation or from 24 or 48 hours after encapsulation).
